# Genus-Wide Pan-Genome Analysis of *Populus* bZIP Transcription Factors with Reanalysis of Public Salt-Stress Transcriptomes

**DOI:** 10.3390/plants15172600

**Published:** 2026-08-26

**Authors:** Qi Lin, Yayu Guo, Hou-Ling Wang

**Affiliations:** 1 State Key Laboratory of Tree Genetics and Breeding, School of Biological Sciences and Technology, Beijing Forestry University, Beijing 100083, China; linqi0207@bjfu.edu.cn; 2 Oak Research Center, College of Forestry, Beijing Forestry University, Beijing 100083, China

**Keywords:** *Populus*, bZIP transcription factor, pan-genome, gene duplication, transposable element, salt stress, RNA-seq

## Abstract

Basic leucine zipper (bZIP) transcription factors regulate plant development and stress responses, but their genus-wide diversity in *Populus* remains unclear. We analyzed 19 *Populus* genomes and retained 1764 bZIP proteins, including 21 independent new loci and four annotation corrections. Of these, 1762 were assigned to 79 orthologous gene groups (OGGs), comprising 43 core, 20 soft-core, 15 shell and one cloud OGG, of which 59 showed copy-number variation. Phylogenetic analysis assigned 74 representative pangenes to 13 subfamilies, with five remaining unclassified and motif patterns differing among subfamilies. Whole-genome duplication (WGD)/segmental duplication accounted for 81.0% of OGG-assigned proteins and contributed predominantly to the conserved component. Although 72.2% of bZIP proteins overlapped a transposable element within the gene body or 2-kb flanks, this proportion was modestly lower than in matched non-bZIP genes, and copy-number-variable OGGs showed no greater TE coverage than invariant OGGs. Among retained homologous comparisons, 97.6% had Ka/Ks ≤ 1, supporting predominant purifying selection. Across the heterogeneous public salt-stress RNA-seq datasets analyzed, no OGG showed a significant, directionally concordant response in at least two *Populus* taxa. These results reveal a conserved bZIP framework shaped mainly by ancient duplication alongside variable genomic contexts and transcriptional responses.

## 1. Introduction

The genus *Populus* comprises an important group of forest trees in the Northern Hemisphere and includes numerous fast-growing species of considerable ecological and economic value [1,2]. Because of their rapid growth and amenability to vegetative propagation, poplars are widely used as a model system for investigating woody plant development and environmental adaptation [1]. *Populus* species occupy diverse habitats across the Northern Hemisphere and exhibit extensive genetic diversity and adaptive differentiation [3]. A recently constructed *Populus* super-pan-genome further revealed widespread structural and gene-content variation within the genus [3]. These resources provide a genomic foundation for investigating the retention, copy-number variation, and functional divergence of key regulatory gene families at the genus level.

Basic leucine zipper (bZIP) proteins constitute a conserved yet functionally diverse class of plant transcription factors. The approximately 16-amino-acid basic region contains the highly conserved N-X_7_-R/K motif and a nuclear localization signal and recognizes A-box, C-box, G-box, and abscisic acid-responsive elements (ABREs) centered on an ACGT core; the adjacent leucine zipper consists of heptad repeats of leucine or other hydrophobic residues and mediates dimerization through coiled-coil formation [4]. Selective homo- or heterodimerization generates distinct bZIP combinations, thereby altering DNA-binding specificity and transcriptional activity [4,5]. Comparative phylogenetic analyses suggest that the green-plant bZIP family arose from four ancestral genes and subsequently diversified into 13 major subfamilies in angiosperms [6]. Functionally characterized plant bZIP proteins participate in photomorphogenesis, organ and tissue differentiation, seed maturation, carbon, nitrogen, and energy metabolism, hormone signaling, pathogen defense, and abiotic-stress responses [4,5,6]. Salt stress is prevalent in natural habitats and constrains poplar growth and environmental adaptation by disrupting osmotic and ionic homeostasis and inducing oxidative damage [7]. As key links between stress signals and downstream gene expression, plant bZIP proteins are widely involved in salt-stress responses. For example, group A AREB/ABF proteins act downstream of abscisic acid (ABA)-dependent SNF1-related protein kinase 2 (SnRK2) signaling to regulate stress-responsive genes, whereas members of groups C and S coordinate carbohydrate and energy status with stress responses [8,9]. In poplar, *PagbZIP75* increases salt tolerance through ABA-associated regulation and reduced reactive oxygen species accumulation [10]. Elucidating salt-responsive regulation within the *Populus* bZIP family is therefore important for understanding stress adaptation in poplars.

With the accumulation of high-quality plant genomes, bZIP families have been systematically characterized in *Arabidopsis*, rice, wheat, and several woody plants [4,11,12,13]. Most existing studies, however, rely on a single reference genome. Such analyses may fail to capture presence/absence variation (PAV) and copy-number variation (CNV) within or among closely related species and are sensitive to assembly and annotation completeness, limiting their ability to represent lineage-specific divergence [14,15]. By integrating genomes from multiple individuals or related species, pan-genome analysis can distinguish consistently retained family members from those with variable distributions at the orthologous-group level, providing a framework for resolving complete family composition and interspecific variation [14]. Recent pan-genome studies of the barley bHLH, cucurbit and tea bZIP, and soybean NAC families have resolved conserved and variable components that are obscured in single-reference analyses and linked gene-family diversity to PAV, CNV, duplication mode, and expression divergence [16,17,18,19]. A population-scale pangenome of the filamentous fungus *Neurospora crassa* similarly revealed lineage-associated presence/absence variation in several stress-related gene families, including bZIP factors [20]. Nevertheless, studies of the *Populus* bZIP family have largely focused on member identification and expression analysis in individual species, leaving its genus-wide retention, copy-number variation, and underlying evolutionary mechanisms incompletely resolved.

Here, we integrated genomic resources from 19 *Populus* genomes to systematically identify bZIP family members. Pan-genome classification, phylogenetic reconstruction, and conserved-motif analysis were used to characterize family composition and structural divergence across the genus. We further examined CNV, duplication mode, chromosomal synteny, neighboring transposable elements (TEs), and selection pressure to investigate the origins of conserved and variable components. In addition, we integrated salt-stress RNA sequencing (RNA-seq) datasets from six *Populus* taxa to evaluate transcriptional support for the retained supplementary models and compare salt-responsive patterns among taxa, OGGs, and pan-genome classes. Together, these analyses characterize genus-wide diversity in the *Populus* bZIP family at the levels of genomic retention, structural evolution, and stress-responsive expression, providing a basis for prioritizing candidate regulators of salt adaptation.

## 2. Results

### 2.1. Identification of bZIP Genes and Construction of Orthologous Gene Groups Across 19 Populus Genomes

We initially screened 19 *Populus* genomes, representing 18 species and one variety, and identified 1739 bZIP models in the existing genome annotations. Using BITACORA, we additionally identified 52 candidate records. Of these candidates, six represented complete gene models at the initial assessment, whereas homology-guided gene-model reconstruction generated complete models with canonical PF00170 domains for another 23. Fifteen candidate records overlapped or were derived from existing annotations, including seven split-derived fragments, four annotation-corrected models, three redundant reannotations, and one insufficiently supported alternative ORF. Based on model completeness, domain evidence, overlap with existing annotations, and cross-species homology support, 25 models were retained, comprising 21 independent new loci and four annotation-corrected models at existing loci. The remaining 27 records were excluded from the bZIP dataset used for subsequent analyses, including 14 unresolved bZIP-homologous fragments, seven split-derived fragments originating from five annotated transcripts, two putatively coding-disrupted loci, three redundant reannotations, and one alternative ORF lacking sufficient support (Supplementary Table S1).

We next searched same-species public transcript resources for the 25 retained models. Sequence-accessible EST, TSA, or submitted non-RefSeq mRNA/cDNA records were available for 22 models; the remaining three could not be evaluated because no such sequences were available for their respective species. Among the 22 evaluable models, GWHAAYU00000012_exon20_exon6 from *P. euphratica* received partial support from EST AJ779048.1, with 96.536% nucleotide identity and 74% CDS query coverage. Two additional models produced short local matches that did not meet the criteria for locus-level transcript support, whereas no significant same-species match was detected for the remaining 19 models. Given the uneven coverage of public transcript resources among *Populus* species, the absence of a matching record was not used as the sole basis for excluding an otherwise structurally supported model. Of the 25 retained supplementary models, 12 were represented in the available RNA-seq datasets and were therefore evaluable for transcriptional support; eight of these reached TPM ≥ 1 in at least one sample (Supplementary Table S1).

**Figure 1:**
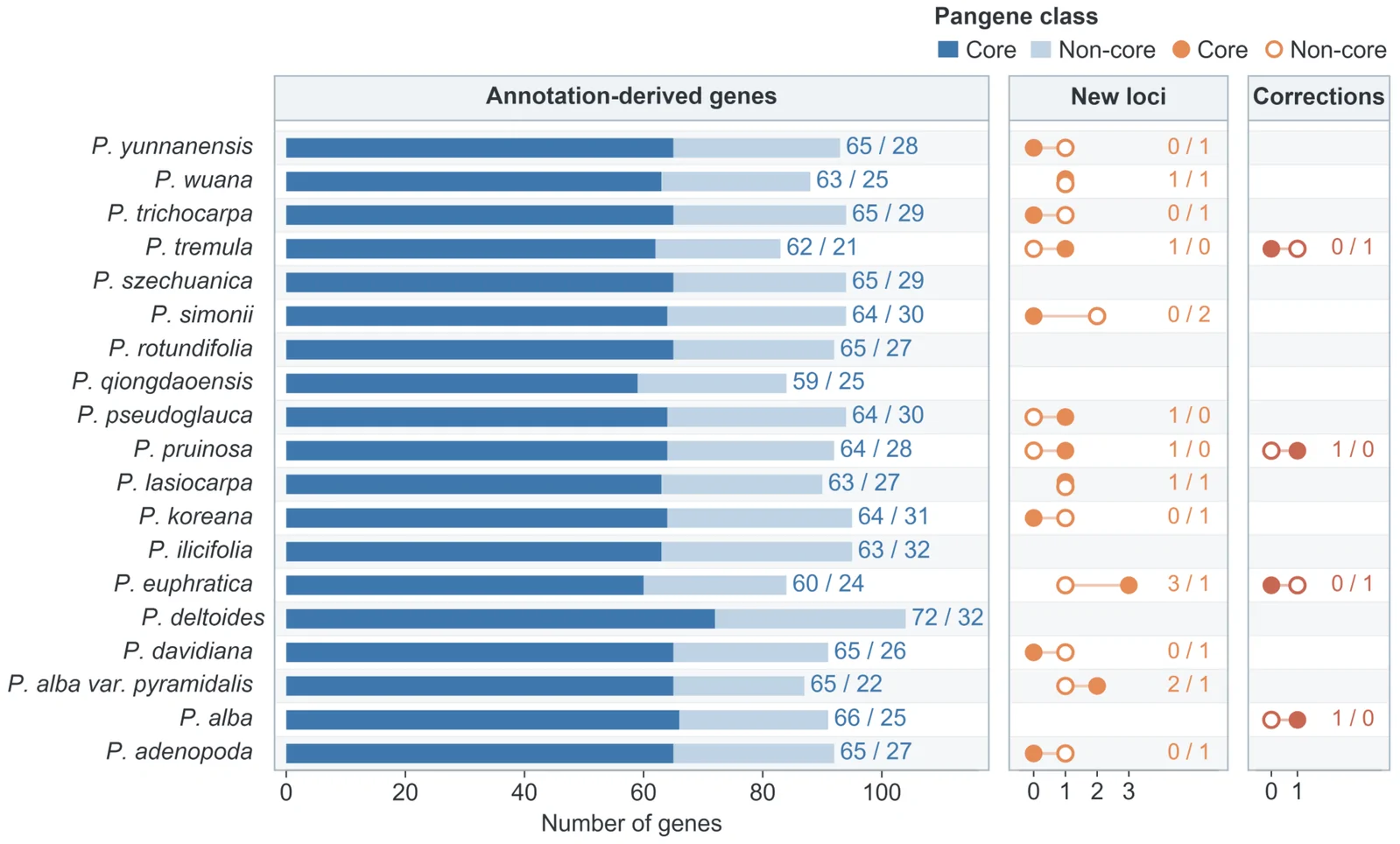
Distribution and source composition of *Populus* bZIP genes across 19 genomes. Horizontal stacked bars show the numbers of bZIP genes obtained from existing genome annotations and assigned to core (dark blue) or non-core (light blue) pangenes in each genome; values at the bar ends indicate core/non-core gene counts. The middle and right panels summarize retained independent new loci and annotation corrections at existing loci, respectively. Filled and open markers denote genes assigned to core and non-core pangenes, respectively, and adjacent labels report the corresponding core/non-core counts.

The resulting dataset comprised 1764 bZIP protein sequences, including 1739 proteins from existing genome annotations and the 25 retained models. Across the 19 genomes, the bZIP family contained an average of 92.8 members, with family size ranging from 85 to 104. *P. tremula* and *P. qiongdaoensis* contained the fewest members (85 each), whereas *P. deltoides* contained the most (104). The 104 *P. deltoides* genes were assigned to 70 of the 79 OGGs, of which 27 contained multiple *P. deltoides* copies. Thus, the larger family size in *P. deltoides* primarily reflected higher copy numbers within shared OGGs rather than a broader OGG repertoire. The remaining 16 genomes contained 90–96 members, indicating that bZIP family size was relatively consistent across most *Populus* genomes. The 25 retained models occurred in 14 genomes, with one to five models per genome; *P. euphratica* contained the largest number (five) (Figure 1; Supplementary Tables S1 and S2).

**Figure 2:**
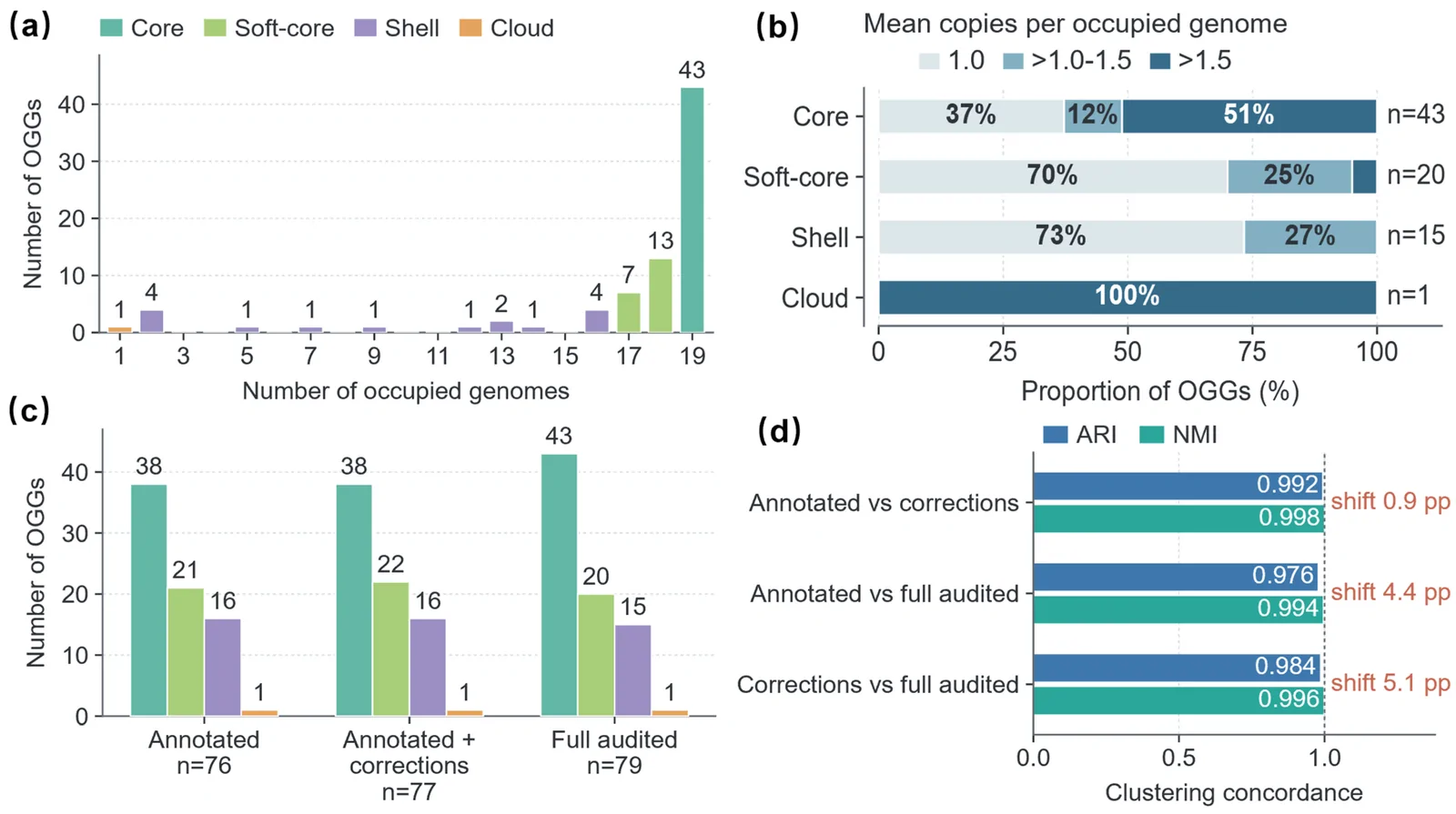
Pan-genome distribution, copy-number variation and input sensitivity of *Populus* bZIP orthogroups. (**a**) Occupancy spectrum of the 79 bZIP orthogroups (OGGs) in the full dataset across 19 *Populus* genomes. Bars show the number of OGGs detected in each number of genomes and are coloured according to pan-genome conservation class. (**b**) Copy-number composition within each conservation class. OGGs were grouped according to their mean number of gene copies per occupied genome (1.0, >1.0–1.5 or >1.5); percentages indicate the proportion in each copy-number category, and n denotes the number of OGGs. (**c**) Sensitivity of OGG classification to retained-model inclusion. “Annotation-derived” contains the 1739 proteins from existing genome annotations, “Annotations + corrections” additionally includes four annotation-corrected models, and “Full dataset” further includes 21 independent new loci. Bars show the numbers of core, soft-core, shell and cloud OGGs in each dataset. (**d**) Concordance of gene clustering among the three datasets, quantified using the adjusted Rand index (ARI) and normalized mutual information (NMI). Values approaching 1 indicate highly similar clustering. “Shift” denotes the maximum absolute percentage-point difference in the proportion of any conservation class between the two datasets being compared. The two proteins not assigned to an OGG were excluded from OGG-level analyses.

At the OGG level, 1762 of the 1764 bZIP proteins were assigned to 79 OGGs, whereas two remained unassigned by OrthoFinder. Locus-level assessment showed that *P. euphratica* Poeup15095.t1 encoded a complete open reading frame with a single canonical PF00170 domain. Although 18 closely related reference proteins were screened, all recovered longer models contained three to five frameshifts and therefore failed the coding-integrity assessment. *P. qiongdaoensis* Poqio15123.t1 also contained a complete open reading frame, but its 674-aa protein carried two canonical PF00170 domains, and its coding region contained an exact 779-bp tandem repeat. Homology-guided analysis recovered a temporary 423-aa alternative model, QIOS000007, which was supported by homologous mappings from six external *Populus* species and assigned to the corresponding orthogroup in a diagnostic OrthoFinder analysis. However, approximately 5.5 million PacBio records and six Illumina libraries provided no junction-spanning evidence that could unambiguously distinguish the original and alternative models. We therefore did not adopt the temporary alternative model. Instead, the two original proteins were retained as bZIP family members but excluded from pan-genome classification and subsequent OGG-level analyses (Supplementary Table S3). Based on their distribution across the 19 genomes, the 79 assigned OGGs were classified into 43 core (54.4%), 20 soft-core (25.3%), 15 shell (19.0%), and one cloud OGG (1.3%). Notably, all 43 core OGGs were present in all 19 genomes. Among the 20 soft-core OGGs, 13 were present in 17 genomes and seven in 18 genomes. Shell OGGs occurred in 3–15 genomes, whereas the single cloud OGG was restricted to one genome (Figure 2a). The number of OGGs also varied among genomes: *P. tremula* contained the fewest, with 64 OGGs, whereas *P. koreana* and *P. simonii* each contained the highest number, with 74 OGGs (Supplementary Tables S4 and S5).

The mean copy number per occupied genome also differed among pan-genome classes. Of the 43 core OGGs, only 16 (37.2%) had a mean copy number of 1, five (11.6%) had values greater than 1 but not exceeding 1.5, and 22 (51.2%) had values greater than 1.5. By comparison, 70.0% of soft-core OGGs (14/20) and 73.3% of shell OGGs (11/15) had a mean copy number of 1. A mean copy number greater than 1.5 was observed in only one soft-core OGG and in no shell OGG. The single cloud OGG also had a mean copy number greater than 1.5, although no broader inference can be drawn from a class represented by only one OGG (Figure 2b). In addition, 59 of the 79 OGGs (74.7%) exhibited copy-number variation across the 19 genomes. Of the remaining 20 OGGs with invariant copy numbers, 16 were consistently single-copy and four were consistently present in two copies. Thus, variation among the 19 *Populus* genomes involved both differential OGG retention and copy-number variation within shared OGGs (Supplementary Table S6).

To assess whether apparent absences in soft-core and shell OGGs might be attributable to incomplete genome annotation, we conducted an assembly-level TBLASTN audit of 167 missing genome–OGG combinations. Strong target-homologous genomic hits were identified for 99 combinations, whereas 41 produced intermediate-strength hits. In 24 combinations, the best hit corresponded to a bZIP paralog already assigned to another OGG, while the remaining three produced only weak or partial hits (Supplementary Table S7). These findings suggest that some apparent absences may reflect missing or incomplete gene models. However, TBLASTN similarity alone is insufficient to establish that the corresponding regions encode complete bZIP proteins; these loci were therefore not added to the family dataset or used to revise OGG occupancy calls. The 19 genome resources differed in assembly contiguity and structural-annotation provenance. Sixteen assemblies were chromosome-scale and three were scaffold-level drafts, with gene-set BUSCO completeness ranging from 89.3% to 99.2%. Six genomes were annotated using the unified pipeline of Shi et al. [3], whereas the remaining gene sets were derived from their source studies or database releases and differed in annotation software and supporting evidence (Supplementary Table S8). These differences may have contributed to some apparent absence calls.

To assess whether retained-model inclusion affected OGG inference and pan-genome classification, we constructed two progressively restricted datasets and repeated the analysis using identical parameters. When only the 1739 originally annotated proteins were analyzed, 1737 proteins were assigned to 76 OGGs, comprising 38 core, 21 soft-core, 16 shell, and one cloud OGG. After retaining the four annotation-corrected models, 1741 proteins were assigned to 77 OGGs, comprising 38 core, 22 soft-core, 16 shell, and one cloud OGG. The full dataset contained 43 core, 20 soft-core, 15 shell, and one cloud OGG (Figure 2c). Clustering of genes shared among the three datasets remained highly concordant, with pairwise adjusted Rand indices of 0.976–0.992 and normalized mutual information values of 0.994–0.998; the maximum change in the proportion of any pan-genome class ranged from 0.9 to 5.1 percentage points (Figure 2d). Therefore, although the inclusion of supplementary models altered the boundaries and conservation classifications of some OGGs, the overall clustering of shared genes and the predominance of core OGGs in the pan-genome composition remained stable. As an additional threshold-sensitivity check, lowering the soft-core cutoff from 17 to 16 genomes did not alter the classification because no OGG was present in exactly 16 genomes.

### 2.2. Populus bZIP Pangenes Show Subfamily-Specific Phylogenetic and Motif Patterns

**Figure 3:**
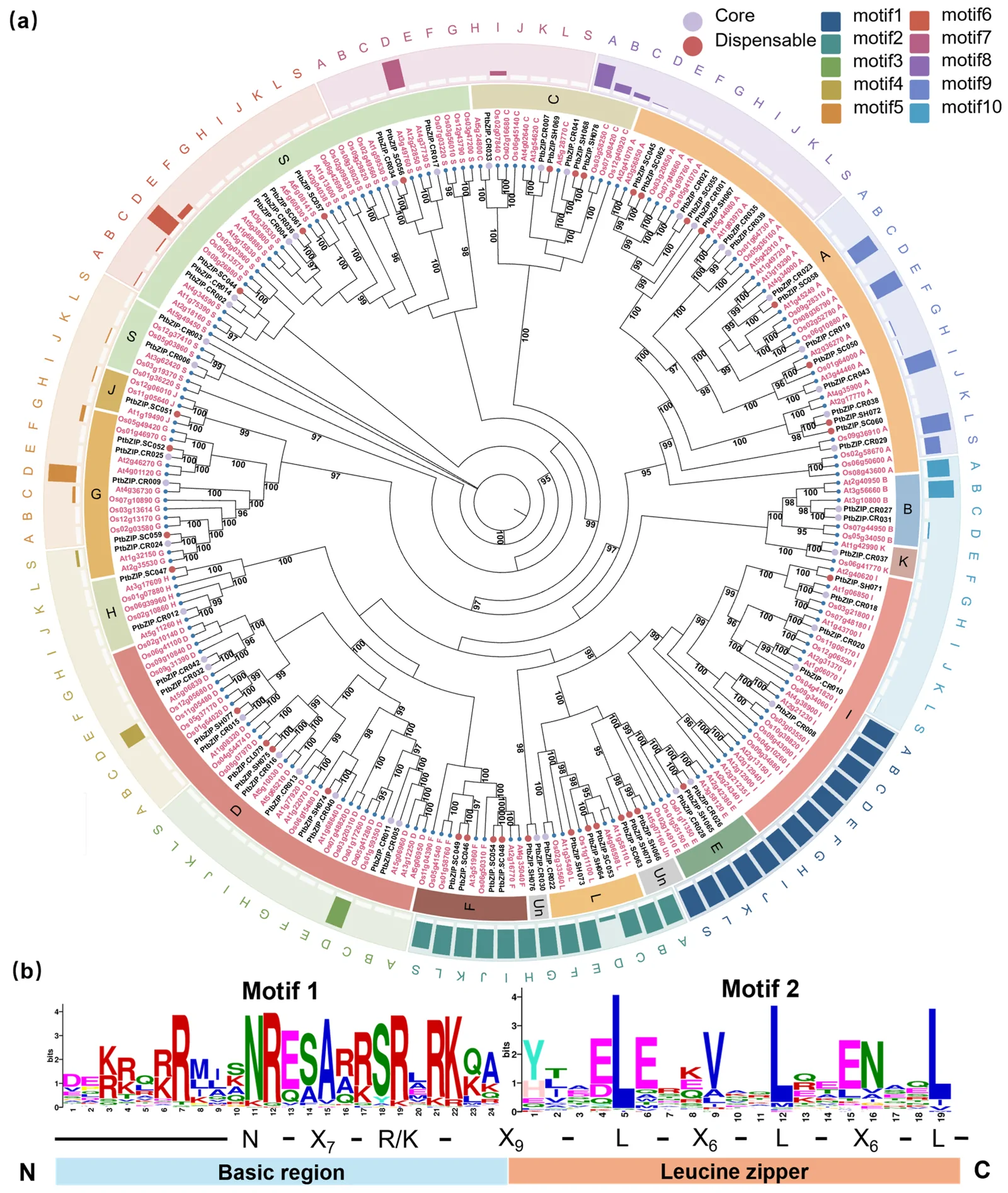
Phylogenetic classification and conserved motif architecture of *Populus* bZIP pangenes. (**a**) Maximum-likelihood phylogeny of 79 representative PtbZIPs and 164 reference bZIP proteins from *Arabidopsis thaliana* and *Oryza sativa*. Coloured sectors denote subfamilies A–S; Un indicates unclassified sequences. Branch labels show UFBoot support ≥95% from 1000 replicates. Tip circles distinguish core and dispensable pangenes, with the latter comprising soft-core, shell and cloud classes. Outer bars show the within-subfamily frequencies of motifs 1–10 among the 1695 proteins assigned to the 13 classified subfamilies. The 67 proteins belonging to five unclassified OGGs are not displayed in the outer ring and are summarized separately in Supplementary Table S9a. (**b**) Sequence logos of motifs 1 and 2 mapped onto the bipartite bZIP domain. Motifs 1 and 2 are shown schematically rather than to scale; their adjacent placement does not imply sequence contiguity; representative MAST coordinates are provided in Supplementary Table S9b. Motif 1 contains the conserved N-X_7_-R/K-X_9_ basic-region signature, whereas motif 2 shows an L-X_6_-L-X_6_-L leucine-heptad-like pattern associated with the leucine zipper.

To characterize the phylogenetic relationships of bZIP genes across the 19 *Populus* taxa, we analyzed 79 representative *Populus* sequences together with reference bZIP proteins from *Arabidopsis* and rice. Of the 79 *Populus* pangenes, 74 were assigned to 13 established bZIP subfamilies [6], whereas five remained unclassified (Un), indicating broad conservation of the subfamily framework across species. Group A was the largest subfamily, containing 17 pangenes, followed by groups D and S, each containing 12, whereas groups J and K each contained only one pangene (Figure 3a; Supplementary Table S5). Pan-genome-class composition also differed among subfamilies. Core pangenes accounted for the largest proportion in groups A (9/17), D (8/12), and S (8/12). All pangenes in groups B and K were core, whereas all four group F pangenes were soft-core. MEME analysis of the 79 OGG representative proteins identified 10 conserved motifs, which were subsequently surveyed across all 1764 bZIP proteins using MAST (Supplementary Table S9a). Motifs 1 and 2 were broadly distributed and were detected in all 13 classified subfamilies. At the pangene level, motifs 1 and 2 occurred in 76 and 66 of the 79 representatives, respectively. At the gene level, motif 1 was detected in 1754 proteins (99.4%), with frequencies ranging from 97.5% to 100% among the classified subfamilies. Motif 2 was detected in 1462 proteins (82.9%) but showed greater variation among subfamilies. It occurred in 87.6–100% of the proteins in the 12 subfamilies other than group D, compared with only 14.5% in group D. The consensus sequence of motif 1 contained the characteristic N-X_7_-R/K signature of the basic region, whereas motif 2 displayed an L-X_6_-L-X_6_-L pattern consistent with the heptad organization of the leucine zipper at the whole-family level (Figure 3b). Other motifs showed stronger subfamily preferences. Motif 3 was restricted to group D and occurred in 97.3% of its proteins, while motifs 5, 6, and 7 were also concentrated in this group, with frequencies of 96.1%, 96.5%, and 98.4%, respectively. Motifs 8 and 10 were prevalent in group A, occurring in 80.6% and 75.6% of its proteins, respectively, whereas motif 10 was also frequent in group B (87.2%). Motif 9 was widely retained in groups C, E, I, and L (91.4–100%) but occurred at a lower frequency in group S (52.9%) (Figure 3a; Supplementary Table S9a).

Notably, MAST detected motif 2 in only 37 of the 255 *Populus* group D bZIP proteins (14.5%), and none of these hits overlapped the PF00170 alignment region or domain envelope, indicating that motif 2 does not reliably delineate the leucine-zipper region in this subfamily. Further assessment of heptad periodicity based on the PF00170 alignment identified the predefined first d position in 250 proteins, of which 249 (99.6%) contained hydrophobic residues at three or more of the five fixed d positions spaced at seven-residue intervals. All 26 group D reference proteins from *Arabidopsis* and rice also met this criterion. The proportion of hydrophobic residues was significantly higher at the fixed d positions than at non-a/d positions within the same aligned region (one-sided paired Wilcoxon signed-rank test, *W* = 31,375, *p* = 1.24 × 10^−43^). Based on the level of sequence support, 79 *Populus* proteins were classified as strong, 170 as moderate, and one as limited, whereas five could not be evaluated because their PF00170 alignments did not extend to the first d position (Supplementary Table S10). Thus, the low detection rate of motif 2 in group D more likely reflects divergence of its leucine-zipper sequences from the family-wide motif consensus than widespread loss of this structure. Nevertheless, these results provide only sequence-based evidence of heptad hydrophobic periodicity and do not substitute for experimental validation of coiled-coil formation or protein dimerization.

To compare copy-number variation and known biological functions among subfamilies, we calculated the number and proportion of copy-number-variable OGGs in each subfamily and compiled experimental evidence from *Arabidopsis* and rice homologs assigned to the same subfamily (Table 1; Supplementary Tables S6 and S11). Of the 74 OGGs assigned to the 13 subfamilies, 55 (74.3%) showed copy-number variation across the 19 *Populus* genomes. Groups A, S, and D contained the largest numbers of copy-number-variable OGGs, comprising 12 of 17 (70.6%), 9 of 12 (75.0%), and 8 of 12 OGGs (66.7%), respectively. All OGGs assigned to groups F, G, H, J, and L were copy-number-variable, although these proportions should be interpreted cautiously given the relatively small sizes of these subfamilies. Group K was the only subfamily in which no copy-number-variable OGG was detected (0/1). Functionally characterized representatives of groups S, D, and A participate in low-energy- and sugar-signal-mediated metabolic regulation, immunity and floral development, and ABA-dependent seed development and abiotic-stress responses, respectively [8,9,21,22,23]. Group I members are associated with vascular development, MAPK responses, and male gametophyte development, whereas group G members contribute to photomorphogenesis and chloroplast establishment [5,24]. Thus, copy-number variation was broadly distributed across subfamilies with diverse biological functions rather than concentrated within a specific functional branch of the bZIP family.

### 2.3. Synteny, Duplication Modes, Transposable Elements, and Evolutionary Patterns of bZIP Genes Across 19 Populus Genomes

**Figure 4:**
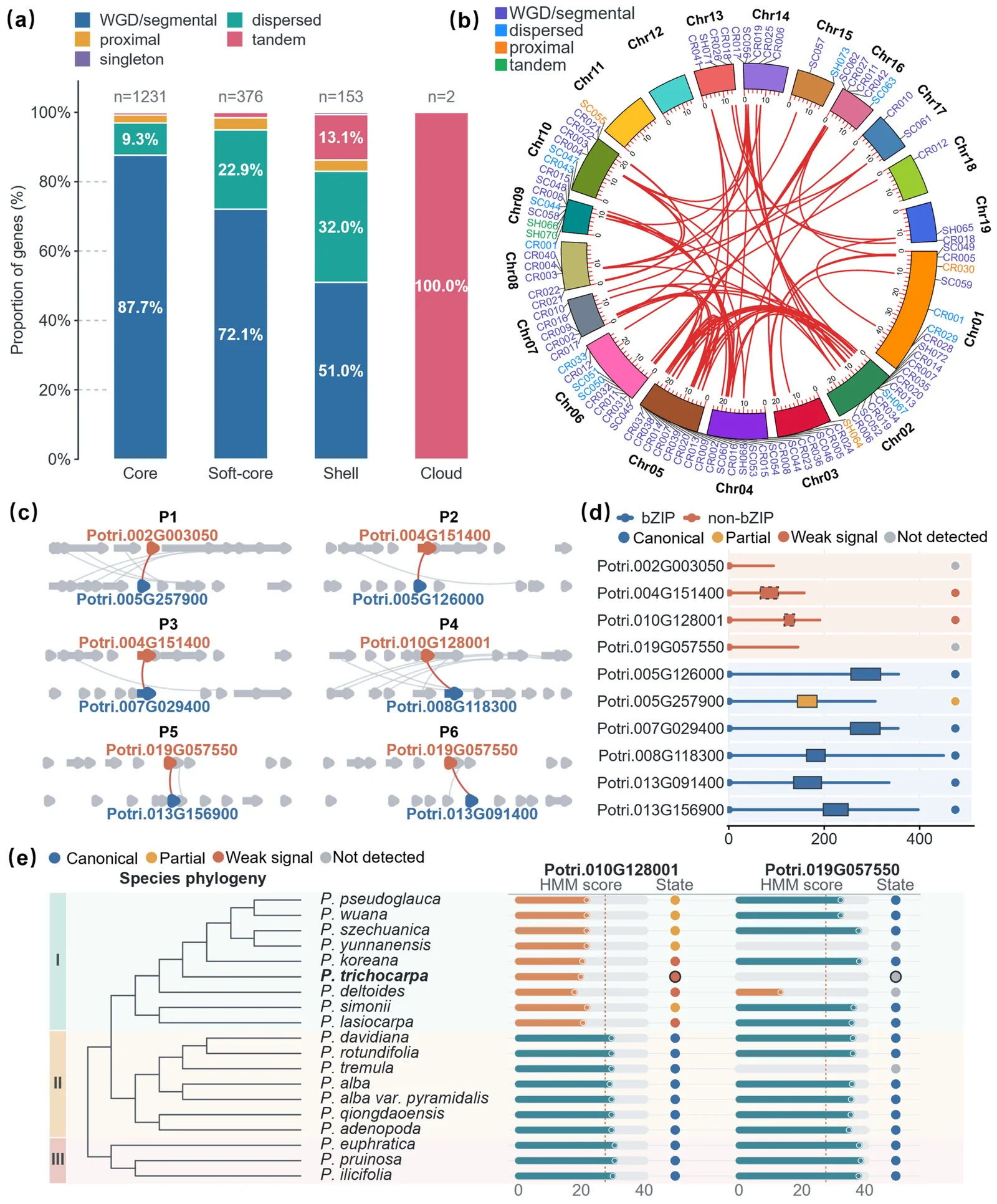
Duplication patterns, chromosomal synteny and PF00170 domain variation in *Populus* bZIP genes. (**a**) Relative frequencies of WGD/segmental, dispersed, proximal, tandem and singleton duplication among bZIP genes in the core, soft-core, shell and cloud pan-genome classes across 19 *Populus* genomes. Bars are normalized to 100%; numbers above the bars indicate sample sizes, and values within the bars indicate the displayed percentages. (**b**) Chromosomal distribution of the 95 *P. trichocarpa* bZIP genes and 72 strict bZIP–bZIP syntenic pairs. Gene labels are coloured according to duplication mode, red curves represent MCScanX-derived syntenic pairs, and chromosome blocks are distinguished by colour. (**c**) Local microsynteny surrounding six MCScanX relationships between confirmed bZIP genes and four candidate loci outside the final bZIP gene set. Blue and orange genes denote confirmed bZIP anchors and candidate loci, respectively; grey arrows represent neighbouring genes, red curves indicate the focal MCScanX relationships, and light-grey curves indicate additional anchors within the corresponding syntenic blocks. (**d**) Protein architecture and PF00170 evidence for the four candidate loci and their six syntenic bZIP anchors. Horizontal lines represent protein length, and rectangles indicate the positions of detected PF00170 matches; weak profile matches are shown with dashed outlines. Blue and orange denote confirmed bZIP anchors and external candidate proteins, respectively. Coloured circles indicate canonical, partial, weak-signal and not-detected PF00170 states. (**e**) PF00170 HMM scores and domain states for homologues of *Potri.010G128001* and *Potri.019G057550* mapped onto the published phylogeny of 19 *Populus* taxa. The phylogenetic topology and branch lengths were retained from Shi et al. [3], with shaded backgrounds indicating Clades I–III. Teal and orange bars indicate HMM scores at or above and below the PF00170 gathering threshold, respectively, and dashed vertical lines mark GA = 27.6. Coloured circles indicate the corresponding domain states. *P. trichocarpa* is highlighted in bold italics, with its state symbols outlined.

To determine the duplication origins of the *Populus* bZIP family, we conducted MCScanX-based intragenomic synteny analyses separately for the 19 genomes and classified the 1762 OGG-assigned bZIP proteins into five duplication modes. WGD/segmental duplication was predominant (1428 proteins, 81.0%), followed by dispersed (249, 14.1%), proximal (46, 2.6%), tandem (37, 2.1%), and singleton duplication (2, 0.1%) (Figure 4a; Supplementary Table S12a,b). Among the three larger pan-genome classes, the proportion of WGD/segmental duplicates decreased from 87.7% in core proteins (1079/1231) to 72.1% in soft-core proteins (271/376) and 51.0% in shell proteins (78/153). Conversely, the proportion of dispersed duplicates increased from 9.3% in core proteins to 22.9% in soft-core proteins and 32.0% in shell proteins, while tandem duplicates accounted for 0.7%, 1.6%, and 13.1% of these classes, respectively. Both cloud proteins were tandem duplicates (2/2, 100%), although this proportion should be interpreted cautiously because the cloud class contained only two proteins. Duplication mode was significantly associated with pan-genome class in the complete 4 × 5 contingency table (Fisher–Freeman–Halton exact test with 1,000,000 Monte Carlo fixed-margin tables, *p* < 1 × 10^−6^; Cramér’s V = 0.239). A sensitivity analysis combining sparsely represented categories similarly supported the association between duplication mode and pan-genome class (χ^2^ = 166.999, df = 4, *p* = 4.61 × 10^−35^; Cramér’s V = 0.218; Supplementary Table S12c). Repeating these analyses after excluding the 25 retained supplementary models yielded the same conclusion: the four-class Fisher–Freeman–Halton exact test remained significant (*p* < 1 × 10^−6^), as did the collapsed analysis (χ^2^ = 191.85, df = 4, *p* = 2.12 × 10^−40^, Cramér’s *V* = 0.235; Supplementary Table S12c). These results indicate that duplication modes made different relative contributions to the conserved and variable components of the family. However, the modest effect size suggests that duplication mode was not the sole determinant of pan-genome class. To further investigate intragenomic duplication and retention patterns of *Populus* bZIP genes, we selected the *P. trichocarpa* reference genome as a representative. The 95 *P. trichocarpa* bZIP genes were assigned to 72 OGGs and distributed across 17 chromosomes. Chr05 and Chr02 contained 12 and 11 members, respectively, whereas the other chromosomes contained 1–9 members each (Figure 4b). Of these 95 genes, 94 were derived from the original genome annotation, while one independent new locus was located on Chr16; no bZIP genes were identified on Chr11 or Chr12. Chr11 and Chr12 contained 1470 and 1248 annotated genes, respectively, and MCScanX detected syntenic blocks linking Chr11 to Chr01 and Chr04 and Chr12 to Chr15. These results indicate that the absence of bZIP genes from these two chromosomes was unlikely to result from chromosome-wide annotation deficiencies or failure to detect synteny. The six weak PF00170 matches detected on Chr11 and Chr12 had E-values of 7.7 × 10^−3^, 1.3 × 10^−2^, 8.3 × 10^−1^, 3.9, 5.2, and 5.9, all of which exceeded the threshold of 1 × 10^−5^ used to identify bZIP homologues in this study; these sequences were therefore not included in the bZIP gene set (Supplementary Table S13a,b). Analysis of the other 18 *Populus* genomes showed that 64 bZIP genes belonging to 25 OGGs were located on Chr11 or Chr12 in 11 genomes. Each of these 25 OGGs retained at least one *P. trichocarpa* member on another chromosome. Thus, the absence of bZIP genes from Chr11 and Chr12 represents a chromosome-distribution feature of the *P. trichocarpa* reference genome rather than a general pattern across the genus. This pattern may be related to chromosomal rearrangements, changes in the positions of homologous segments, or differential retention among lineages; however, the available evidence is insufficient to demonstrate *P. trichocarpa*-specific loss of bZIP genes.

MCScanX identified 72 strict bZIP–bZIP syntenic pairs involving 74 genes, accounting for 77.9% of the *P. trichocarpa* bZIP genes. Of these pairs, 67 connected different chromosomes and five occurred within the same chromosome, and all 74 genes were classified as WGD/segmental duplicates (Figure 4b). The remaining 21 genes were not incorporated into the strict within-family synteny network and comprised 12 dispersed, four WGD/segmental, three proximal, and two tandem duplicates. Further tracing of MCScanX relationships outside the strict within-family network identified six syntenic connections between confirmed bZIP genes and four candidate loci not included in the bZIP gene set (Figure 4c; Supplementary Table S13c). Integration of local synteny, BLASTP protein similarity, PF00170 HMMER searches, and local TBLASTN alignments revealed varying levels of bZIP-related evidence for these four candidate loci (Figure 4c,d; Supplementary Table S13c). *Potri.010G128001* showed relatively strong protein-sequence and local genomic homology to its syntenic bZIP anchor, but its PF00170 HMM score was only 19.8, below the gathering threshold (GA = 27.6), and it was therefore classified as a weak signal. *Potri.019G057550* likewise showed protein-sequence and local genomic homology, but no PF00170 domain meeting the specified criterion was detected. By contrast, *Potri.002G003050* and *Potri.004G151400* received weaker cross-species support for a canonical bZIP domain, and the available evidence was insufficient to include them in the bZIP family. Accordingly, *Potri.010G128001* may represent a candidate with partial domain divergence or an incomplete gene model, whereas *Potri.019G057550* may represent a candidate affected by domain loss or annotation failure. We further mapped PF00170 states and HMM scores onto the phylogenetic relationships of the 19 *Populus* taxa. Homologues of *Potri.010G128001* comprised 10 canonical, five partial, and four weak-signal states, with all nine taxa in Clade I exhibiting partial or weak-signal states. Homologues of *Potri.019G057550* comprised 15 canonical and four not-detected states, with the four not-detected states distributed across Clades I and II rather than being restricted to a single phylogenetic lineage (Figure 4e; Supplementary Table S13c). These results indicate that domain-retention states at the two candidate loci vary among *Populus* lineages, but they remain insufficient to demonstrate definitive post-duplication asymmetric domain loss and require further investigation.

**Figure 5:**
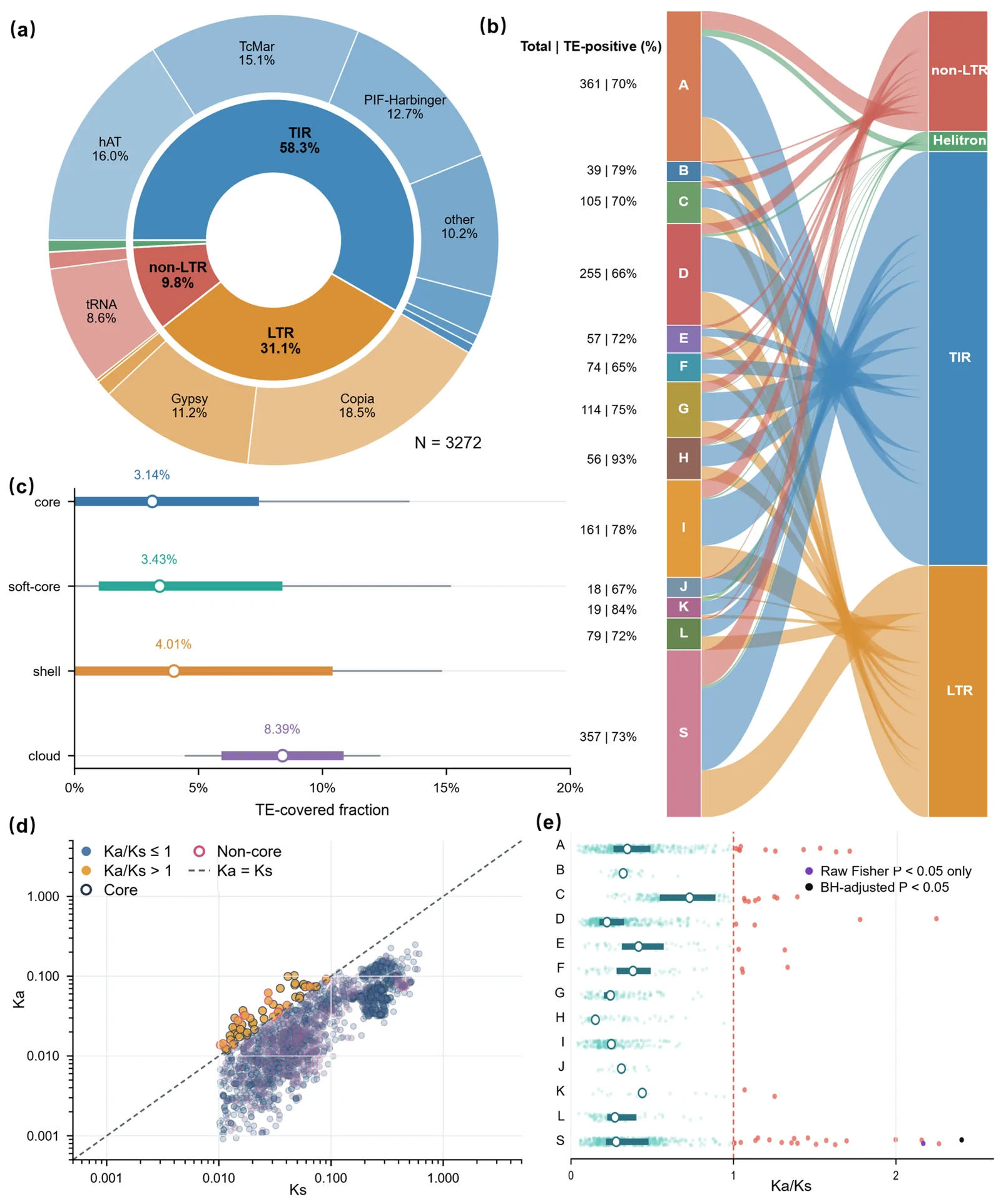
Transposable-element landscape and evolutionary constraints of *Populus* bZIP genes. (**a**) Sunburst representation of 3272 unique transposable-element (TE) copies detected within bZIP gene bodies and their 2-kb upstream and downstream regions. The inner ring shows the proportions of the four major TE classes, and the outer ring shows their principal subclasses. Percentages are calculated from unique TE copies. (**b**) Associations between bZIP subfamilies and the four major TE classes. For each subfamily, Total | TE-positive (%) indicates the number of analysed genes and the percentage containing at least one detected TE, respectively. Ribbon widths represent the numbers of unique gene–TE-class associations and do not imply evolutionary direction. Unclassified genes were excluded from the subfamily-level comparison. (**c**) Distributions of TE-covered fractions within bZIP gene bodies and their 2-kb flanking regions across core, soft-core, shell and cloud genes. Thin lines indicate the 10th–90th percentile range, thick lines indicate the interquartile range, open circles denote medians, and numerical labels show median TE-covered fractions. (**d**) Relationships between Ka and Ks for retained homologous comparisons. Blue and orange points indicate comparisons with Ka/Ks ≤ 1 and Ka/Ks > 1, respectively; filled and open symbols denote core and non-core comparisons. The dashed line indicates Ka = Ks. (**e**) Ka/Ks distributions across the 13 classified bZIP subfamilies. Pale points show retained pairwise estimates for descriptive purposes. Open circles and thick horizontal lines indicate the median and interquartile range, respectively, of OGG-level median Ka/Ks values. Distinct colours identify pairwise estimates supported by raw Fisher or Benjamini–Hochberg-adjusted *p* < 0.05. The dashed line denotes Ka/Ks = 1. The 52 estimates from unclassified OGGs were excluded; the subfamily-level test used one median value for each of 73 classified OGGs.

To evaluate the relationship between TEs and the local genomic environment of bZIP genes, we quantified TEs overlapping the selected mRNA span, including annotated UTRs, and the 2-kb upstream and downstream windows across all 19 genomes. Of the 1764 final bZIP members, 1274 (72.2%) overlapped at least one TE. Among the 3272 unique TE copies, TIR elements were most abundant (1908, 58.3%), followed by LTR (1017, 31.1%), non-LTR (320, 9.8%), and Helitron elements (27, 0.8%). LTR/Copia was the most common subclass (606/3272, 18.5%), followed by DNA/hAT (524/3272, 16.0%), DNA/TcMar (495/3272, 15.1%), DNA/PIF-Harbinger (415/3272, 12.7%), and LTR/Gypsy (367/3272, 11.2%) (Figure 5a; Supplementary Table S14a). At the subfamily level, TIRs were the most frequently associated class in 12 of the 13 classified subfamilies; group E was the exception, with more gene–TE-class associations involving LTRs than TIRs. The proportions of genes associated with at least one TE were highest in groups H (52/56, 92.9%) and K (16/19, 84.2%), but lower in groups F (48/74, 64.9%) and D (168/255, 65.9%) (Figure 5b; Supplementary Table S14a). Among the 1762 OGG-assigned proteins, the proportions associated with at least one TE were 70.9% (873/1231), 76.3% (287/376), 72.5% (111/153), and 100% (2/2) in the core, soft-core, shell, and cloud classes, respectively. The corresponding median TE-covered fractions were 3.14%, 3.43%, 4.01%, and 8.39%. No significant overall difference was detected among the four pan-genome classes (Kruskal–Wallis test, H = 6.596, *p* = 0.086), indicating that local TE burden did not differ robustly among the classes (Figure 5c; Supplementary Table S14a). To determine whether the observed TE overlap exceeded the local genomic background, we compared the bZIP genes with 1000 sets of non-bZIP genes matched by species, chromosome, transcript length, and relative chromosomal position. The observed TE-positive proportion was modestly lower than the matched-control expectation (72.22% versus a mean of 76.91%; difference = −4.69 percentage points, 95% interval = −6.41 to −3.00; empirical two-sided *p* = 0.001998; Supplementary Table S14b). To examine the relationship between local TE burden and copy-number variation, we further compared aggregate TE coverage between copy-number-variable and invariant OGGs. Copy-number-variable OGGs (n = 59) had a median aggregate TE coverage of 5.18% (IQR, 3.41–7.84%), whereas invariant OGGs (n = 20) had a median of 6.05% (IQR, 4.64–8.16%). The distributions did not differ significantly (Mann–Whitney U = 538, *p* = 0.561; Cliff’s delta = −0.088, 95% CI = −0.366 to 0.198), providing no evidence that copy-number-variable OGGs had greater local TE coverage (Supplementary Table S14d,e).

To assess selection pressure among homologous *Populus* bZIP genes, we constructed anchor-to-member comparisons within individual OGGs. Of the 2497 comparisons evaluated, 2231 were retained after filtering, whereas 266 were excluded: 106 because of undefined or invalid estimates and 160 because Ks was < 0.01. After the undefined or invalid estimates were removed, no additional comparisons were excluded by the Ks > 2, Ka > 2, or Ka/Ks > 10 criteria. Among the retained comparisons, 2178 (97.6%) had Ka/Ks ≤ 1 and only 53 (2.4%) had Ka/Ks > 1; the overall median was 0.290. Among the 2231 retained comparisons, 327 (14.7%) were based on fewer than 10 inferred substitutions, whereas the median number of substitutions was 31, and the median Ks was 0.066. At the pairwise level, core and non-core OGGs contributed 1783 and 448 estimates, respectively; Ka/Ks exceeded 1 in 39 core comparisons (2.2%) and 14 non-core comparisons (3.1%) (Supplementary Table S15a,b). Because comparisons sharing an OGG were not independent, group-level inference was based on one median Ka/Ks value per OGG. Among the 77 OGGs with retained estimates, the 43 core and 34 non-core OGGs had median Ka/Ks values of 0.266 and 0.361, respectively, with no significant difference between the two groups (Mann–Whitney U = 628, *p* = 0.293; Cliff’s delta = −0.141, 95% CI = −0.416 to 0.135; Figure 5d; Supplementary Table S15c,d). OGG-level median Ka/Ks values were below 1 in all 13 classified subfamilies. Among the 73 classified OGGs with retained estimates, these medians showed modest overall heterogeneity among subfamilies (Kruskal–Wallis H = 21.648, df = 12, *p* = 0.0417). Group C had the highest OGG-level median (0.730), followed by groups K (0.439), E (0.416), and F (0.382), although group K was represented by only one OGG. At the pairwise level, most of the 53 estimates with Ka/Ks > 1 occurred in groups S, A, C, and D, which contributed 20, 10, eight, and four comparisons, respectively. However, only two elevated-ratio comparisons had a raw Fisher *p* < 0.05, and only one remained significant after Benjamini–Hochberg correction across all 2231 retained comparisons (Figure 5e; Supplementary Table S15a,d). Thus, the results support predominant purifying constraint across the *Populus* bZIP family. The limited information content of some pairwise estimates and the small number of statistically supported elevated ratios do not support widespread positive selection; the modest OGG-level differences among subfamilies are more appropriately interpreted as variation in selective constraint.

### 2.4. Multispecies Salt-Stress Expression Profiles of Populus bZIP Genes

**Figure 6:**
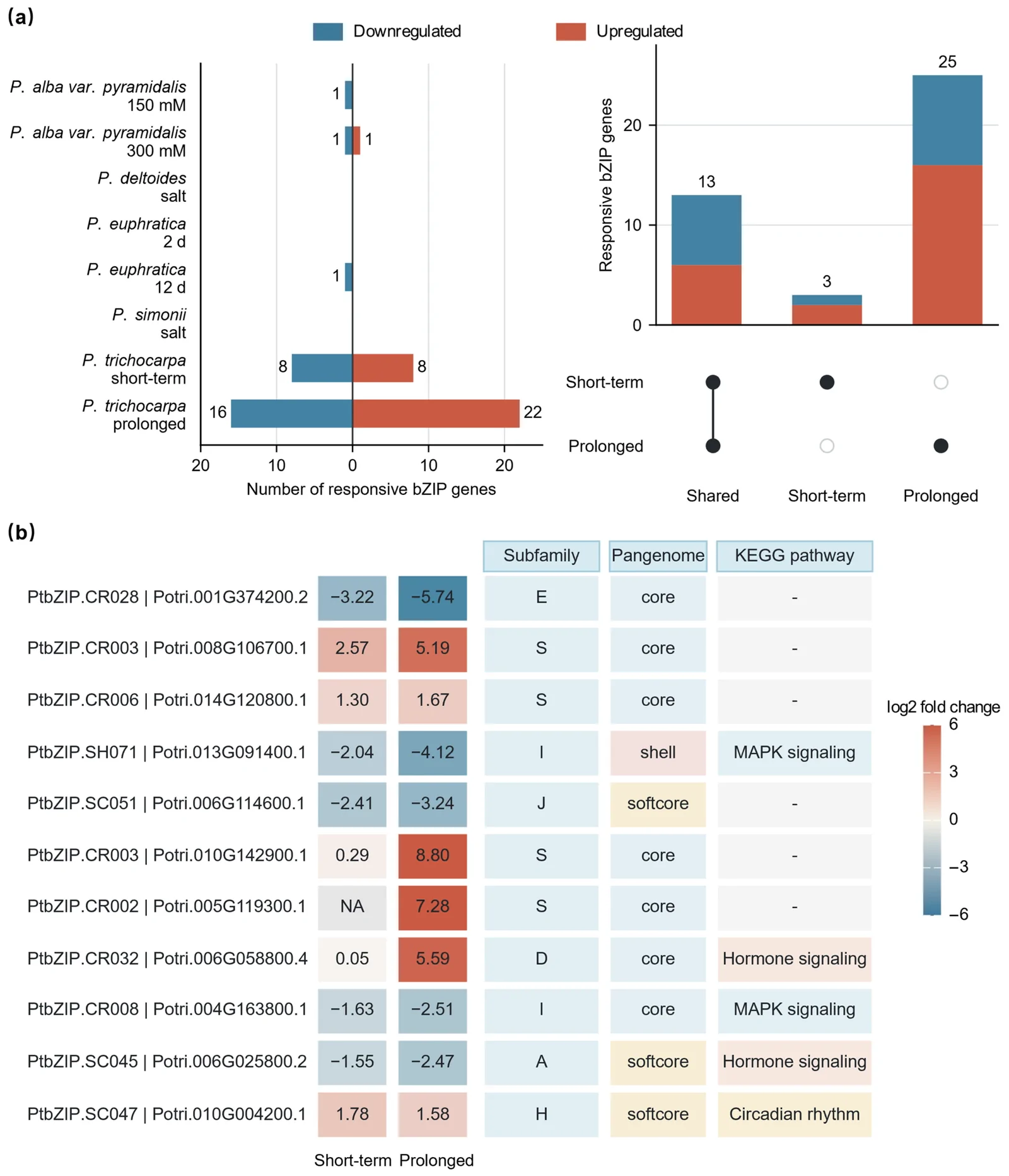
Salt-responsive expression dynamics of *Populus* bZIP genes. (**a**) Numbers of significantly up- and downregulated bZIP genes across eight replicated salt-stress contrasts from five *Populus* taxa (left), and overlap of responsive *P. trichocarpa* genes between short-term and prolonged treatments (right). The short-term and prolonged *P. trichocarpa* contrasts used the same three untreated libraries as controls; accordingly, their overlap represents temporal consistency within a shared-control dataset rather than independent replication. The stacked bars show the numbers of up- and downregulated genes in the shared, short-term-specific, and prolonged-treatment-specific sets. Filled and open circles indicate inclusion and exclusion, respectively, and connecting lines denote treatment-set intersections. Significant responses were defined by |log2 fold change| ≥ 1 and Benjamini–Hochberg-adjusted *p* ≤ 0.05. (**b**) Expression patterns and functional annotations of eleven representative salt-responsive *P. trichocarpa* bZIP genes. Heatmap cells show log2 fold changes under short-term and prolonged treatments; NA indicates that *Potri.005G119300* had zero counts across all libraries included in the short-term contrast and was consequently excluded from the testable set by DESeq2 independent filtering; therefore, no finite log2 fold-change estimate or adjusted *p*-value was returned. Adjacent tracks indicate bZIP subfamily, pan-genome class, and selected KEGG pathway annotations. Blue and coral denote downregulation and upregulation, respectively. The color scale is truncated at −6 and 6, whereas cell labels report the original log2 fold-change values.

To characterize the salt-stress expression profiles of *Populus* bZIP genes, we integrated 53 salt-stress RNA-seq libraries from six taxa (Supplementary Table S16). Of the 25 supplementary models retained after structural curation, 12 were evaluable using the RNA-seq datasets available for the six *Populus* taxa, and 8 reached TPM ≥ 1 in at least one sample. GWHAAYU00000012_exon20_exon6, which received partial support from a same-species EST, was among these eight RNA-seq-supported models. The remaining 13 models could not be evaluated because corresponding taxon-level RNA-seq data were unavailable. These results provide independent transcriptional support for a subset of the structurally retained models, while the transcriptional status of the unevaluated models remains unresolved (Supplementary Table S17a,b). Across the eight replicated salt-stress contrasts, 58 significant bZIP response events were detected, including 31 upregulation and 27 downregulation events (Supplementary Tables S18 and S19d). Across the eight replicated contrasts, 61–77 bZIP genes had finite testable estimates, median library depth ranged from 16.3 to 45.1 million reads, median mapping rate ranged from 85.0% to 98.9%, and each treatment group contained three to five biological replicates. Five contrasts yielded no more than two responsive genes, whereas the *P. trichocarpa* dataset contributed 54 of the 58 significant response events (Supplementary Table S19d). Response magnitude differed markedly among datasets. The 150 mM and 300 mM NaCl contrasts in *P. alba* var. *pyramidalis* contained one and two responsive genes, respectively, and across the two *P. euphratica* contrasts, only one downregulated gene was detected. No gene reached the response threshold in *P. simonii* or *P. deltoides*. By contrast, *P. trichocarpa* showed a broader bZIP response. Short-term treatment affected 16 genes, with eight upregulated and eight downregulated, whereas prolonged treatment affected 38 genes, with 22 upregulated and 16 downregulated. The short-term and prolonged *P. trichocarpa* contrasts used the same three untreated libraries as controls. Thirteen genes were significant and changed in the same direction in both contrasts; this overlap was therefore interpreted as temporal consistency within the dataset rather than as independent replication across treatment stages (Figure 6a).

To examine temporal variation in the salt response of *P. trichocarpa* bZIP genes, we selected 11 genes representing sustained and prolonged-treatment-specific expression patterns (Figure 6b). Eight genes responded significantly at both treatment stages. Among them, *Potri.008G106700*, *Potri.010G004200*, and *Potri.014G120800* were consistently upregulated, whereas *Potri.001G374200*, *Potri.013G091400*, *Potri.006G114600*, *Potri.004G163800*, and *Potri.006G025800* were consistently downregulated. The remaining three genes, *Potri.010G142900*, *Potri.005G119300*, and *Potri.006G058800*, were specifically upregulated under prolonged treatment and showed relatively large expression changes, with log2 fold changes of 5.59–8.80. Of particular interest, *Potri.014G120800* showed sustained upregulation under both short-term and prolonged treatments, with log2 fold changes of 1.30 and 1.67, respectively. Its corresponding gene, PagbZIP75, has been experimentally shown to enhance salt tolerance in poplar through ABA-associated regulation and reduced ROS accumulation [10], providing functional support for the response observed here. Functional annotation further linked *Potri.013G091400* and *Potri.004G163800* to the plant MAPK signaling pathway, *Potri.006G025800* and *Potri.006G058800* to plant hormone signal transduction, and *Potri.010G004200* to the plant circadian rhythm pathway. Together, the sustained response and existing functional evidence highlight *Potri.014G120800* as a priority candidate, while the strong prolonged-treatment-specific induction of *Potri.010G142900*, *Potri.005G119300*, and *Potri.006G058800* supports their inclusion in subsequent functional analyses (Figure 6b; Supplementary Table S18). We next evaluated cross-taxon consistency at the OGG level. Of the 76 assigned OGGs represented in at least one of the six taxon-level expression datasets, 44 showed no significant response and 29 responded significantly in only one taxon. Three OGGs—PtbZIP.CR010, PtbZIP.CR012 and PtbZIP.CR020—responded significantly in two taxa, but each showed opposite response directions between taxa. Thus, no OGG exhibited a significant, directionally concordant salt response in at least two taxa, and robust cross-taxon response patterns could not be inferred from these heterogeneous public datasets (Supplementary Table S19a).

**Figure 7:**
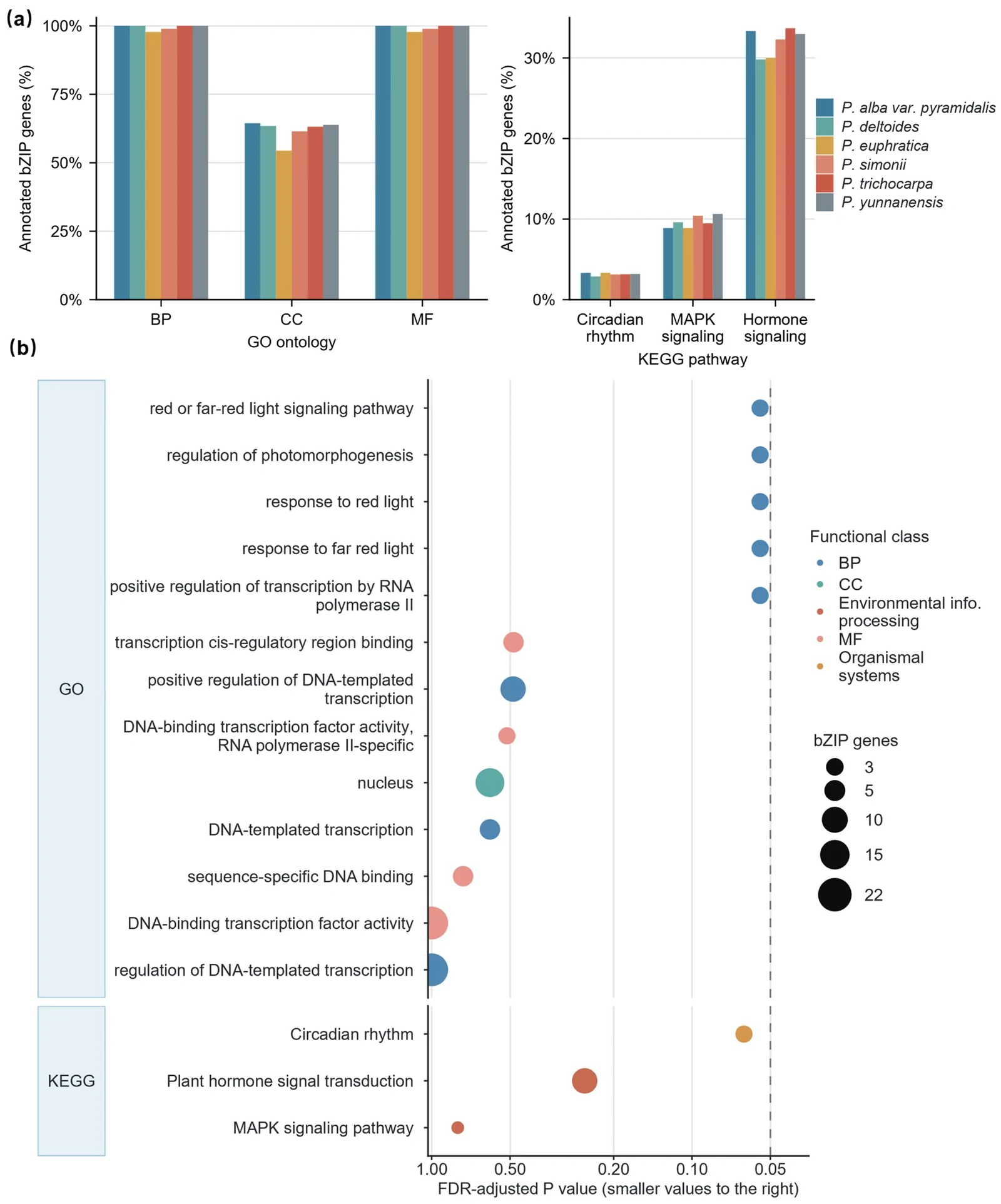
Functional annotation profiles and salt-responsive enrichment trends of *Populus* bZIP genes. (**a**) Proportions of bZIP genes annotated to the three Gene Ontology (GO) domains—biological process (BP), cellular component (CC), and molecular function (MF)—and to the KEGG pathways circadian rhythm–plant, MAPK signaling pathway–plant, and plant hormone signal transduction across six *Populus* taxa. Colored bars denote individual taxa, and percentages were calculated relative to the corresponding taxon-level bZIP catalogue. (**b**) GO and KEGG over-representation analysis of the 22 bZIP genes upregulated under prolonged salt treatment in *P. trichocarpa*, using the 77 bZIP genes with finite log2 fold-change estimates in that contrast as the background. Point positions show Benjamini–Hochberg-adjusted *p*-values on a reversed logarithmic scale, with smaller values plotted to the right. Point sizes indicate the numbers of bZIP genes assigned to each term, and colors denote GO domains or KEGG functional classes. The dashed vertical line marks FDR = 0.05; none of the displayed terms passed this threshold.

To investigate the potential functions of salt-responsive bZIP genes, we assessed functional-annotation coverage and category composition across the six taxon-specific bZIP sets. Annotation coverage for both GO biological process and molecular function ranged from 97.8% to 100.0%, whereas cellular component coverage ranged from 54.4% to 64.4%. Plant hormone signal transduction was the most frequent KEGG category in every taxon, covering 29.8–33.7% of the corresponding bZIP genes, whereas the plant MAPK signaling pathway and plant circadian rhythm accounted for 8.9–10.6% and 2.9–3.3%, respectively (Figure 7a; Supplementary Table S19b). We next performed GO and KEGG over-representation analyses for the salt-responsive gene sets from each replicated contrast. Among these comparisons, nominal evidence of over-representation was detected only for the 22 bZIP genes upregulated under prolonged salt treatment in *P. trichocarpa*, although no GO term or KEGG pathway remained significant after Benjamini–Hochberg correction. Three related GO biological process terms, red or far-red light signaling pathway, regulation of photomorphogenesis, and response to red or far-red light, each contained the same three genes: *Potri.006G251800*, *Potri.010G004200*, and *Potri.018G029500* (3/22 responsive genes versus 3/77 background genes; raw *p* = 0.0211 and adjusted *p* = 0.0547 for each term). The same three genes were assigned to the KEGG pathway circadian rhythm–plant (3/22 versus 3/77; raw *p* = 0.0211, adjusted *p* = 0.0632). Plant hormone signal transduction contained 10 of the 22 upregulated genes but was not over-represented relative to the testable bZIP background (10/22 versus 27/77; adjusted *p* = 0.258) (Figure 7b; Supplementary Table S19c). These results indicate nominal trends involving light signaling, photomorphogenesis, and circadian regulation, rather than statistically significant functional enrichment.

## 3. Discussion

Advances in plant genome sequencing and assembly have established pan-genome analysis as a framework for investigating genetic diversity across species and genera. Using 19 *Populus* genomes, we identified 1764 bZIP proteins, of which 1762 were assigned to 79 OGGs, while two remained unassigned and were excluded from OGG-level analyses. Copy-number variation was detected in 59 of the 79 OGGs across the 19 genomes. The family therefore retained a broadly shared orthologous-group framework while generating genus-wide variation through both differential OGG occupancy and copy-number changes within shared OGGs. Such patterns of genus-wide retention and variation are difficult to resolve using a single reference genome [3,14]. By integrating multiple *Populus* genomes, the pan-genome framework provided a more complete genus-level view of bZIP family composition, copy-number variation and lineage-associated divergence. Sensitivity analyses further showed that, although retained-model inclusion altered some OGG boundaries and conservation assignments, the overall clustering of shared genes remained highly concordant across input datasets.

Locus-by-locus evaluation of the BITACORA-derived candidates showed that the 52 initially identified records could not all be regarded as independent newly predicted genes. Following gene-structure recovery and evidence-based assessment, 21 independent new loci and four annotation-corrected models at existing loci were retained. These 25 supplementary models were not restricted to variable components: 12 were assigned to core OGGs, 10 to soft-core OGGs, and three to shell OGGs. Thus, supplementary identification recovered potentially overlooked members of broadly conserved orthogroups while also improving the representation of variable components among genomes. Of the 12 retained models evaluable using the available RNA-seq data, eight reached TPM ≥ 1 in at least one sample, and one of these also received partial support from a same-species EST, providing independent transcriptional evidence for a subset of the supplementary models. Most retained models lacked matching records in the public EST, TSA, or submitted mRNA/cDNA resources. However, because the coverage of these resources varied among *Populus* species and biological contexts, the absence of a public transcript match could not be interpreted as evidence of transcriptional inactivity or pseudogenization. During long-term plant divergence, gene gain, loss, and differential retention can collectively generate presence/absence variation among species or populations [14]. At the same time, missing gene models or incomplete structural annotations can produce apparent gene absences and directly affect the inference of gene presence or absence [15]. The 19 *Populus* genome resources analyzed in this study differed to some extent in assembly contiguity, structural annotation pipelines, and supporting evidence types, indicating that some absence calls may be influenced by the completeness of the available assemblies and annotations (Supplementary Table S8). Together with the assembly-level TBLASTN audit, these differences suggest that some apparent absences in soft-core and shell OGGs may reflect annotation omissions or assembly limitations rather than genuine gene loss. Integrating cross-genome homology and domain searches with locus-level structural evaluation therefore helps distinguish genuine genomic variation from annotation omissions, reduces biases arising from individual annotation resources, and provides a more reliable basis for resolving gene-family conservation and divergence.

Phylogenetic and motif analyses indicated that pan-genome variation in the *Populus* bZIP family occurred primarily within the established subfamily framework. Of the 79 pangenes, 74 were assigned to 13 established bZIP subfamilies, whereas five remained unclassified. Core, soft-core, and shell members clustered mainly according to their subfamily relationships rather than forming independent branches corresponding to pan-genome classes. This pattern is consistent with the early origin and subsequent evolutionary stability of the major angiosperm bZIP subfamilies [6]. At the structural level, the consensus sequence of motif 1 contained the characteristic N-X_7_-R/K signature of the basic region, whereas motif 2 exhibited leucine periodicity consistent with the heptad repeats of a leucine zipper [5,25]. These motifs correspond to regions involved in DNA binding and protein dimerization, respectively, and their broad distribution across *Populus* bZIP subfamilies further supports conservation of the basic structural framework of this family. Beyond this shared framework, individual subfamilies exhibited preferences for specific motifs, potentially reflecting the differential retention of additional regulatory sequence modules among functional lineages.

In group A, motifs 8 and 10 were detected in 80.6% and 75.6% of the proteins, respectively. Previous studies have shown that the conserved C1-C4 regions of group A AREB/ABF proteins contain protein kinase recognition sites. These sites can be phosphorylated by ABA-activated SnRK2 protein kinases, and their multisite phosphorylation regulates AREB1 transcriptional activity and ABRE-dependent gene expression [21]. The preferential occurrence of motifs 8 and 10 in group A is consistent with the involvement of this subfamily in ABA-mediated seed germination, growth regulation, and responses to abiotic stress [8]. In group D, motifs 3, 5, 6, and 7 were detected in 97.3%, 96.1%, 96.5%, and 98.4% of the proteins, respectively, with motif 3 being restricted to this subfamily. Motif 5 was enriched in glutamine and acidic residues, consistent with the sequence composition of conserved regulatory regions in TGA proteins [22]. Regions outside the bZIP domain of TGA proteins contribute to transcriptional regulation and interactions with proteins such as NPR1 and ROXY, thereby linking TGA factors to salicylic acid signaling and pathogen defense [22,23]. The stable co-occurrence of multiple motifs in group D may therefore reflect the retention of relatively conserved protein-interaction and transcriptional-regulatory regions in addition to the bZIP domain.

Notably, motif 2 was detected in only 14.5% of group D proteins, whereas the PF00170-anchored analysis of heptad periodicity indicated that this subfamily generally retained the characteristic hydrophobic periodicity of the leucine zipper. Thus, the low detection rate of motif 2 does not indicate widespread loss of the leucine zipper in group D, but more likely reflects subfamily-specific sequence divergence in this region from the family-wide motif 2 consensus. Moreover, although the leucine zipper sequences of TGA proteins differ from those of other bZIP subfamilies, these proteins can still form functional homodimers and heterodimers [5,25,26]. Such divergence may preserve the basic capacity for dimerization while altering the properties of the protein-interaction interface, thereby influencing dimer stability, partner selection, and the specificity of downstream transcriptional regulation.

Copy-number patterns further indicated that copy-number variation among genomes was broadly distributed across different subfamilies rather than confined to a single functional lineage. Of the 74 OGGs assigned to 13 subfamilies, 55 (74.3%) exhibited copy-number variation among the 19 genomes. Groups A, S, and D contained the largest numbers of copy-number-variable OGGs, comprising 12 of 17 (70.6%), 9 of 12 (75.0%), and 8 of 12 OGGs (66.7%), respectively. All OGGs assigned to groups F, G, H, J, and L were copy-number-variable, although these proportions may partly reflect the relatively small sizes of these subfamilies. Group K was the only subfamily in which no copy-number-variable OGG was detected (0/1). Functionally characterized members of groups A, S, and D participate in ABA and abiotic-stress responses, sugar and energy metabolism, and defense and salicylic acid signaling, respectively [8,9,23]. The widespread occurrence of CNV across these and other functional branches may provide a genetic basis for the stable maintenance of core biological functions and the diversification of environmental responses through regulatory redundancy and gene-dosage variation. However, because subfamily sizes differ considerably, these patterns alone do not demonstrate adaptive expansion of any particular functional branch.

Meanwhile, duplication-mode analysis provided an evolutionary context for these differences. WGD/segmental duplicates accounted for 81.0% of all OGG-assigned bZIP genes, but their proportion declined from 87.7% in core genes to 72.1% in soft-core genes and 51.0% in shell genes. Tandem duplicates, in contrast, accounted for only 0.7% of core genes but 13.1% of shell genes, whereas dispersed duplicates increased from 9.3% in core genes to 32.0% in shell genes. Both cloud genes were tandem duplicates, although this observation should be interpreted cautiously because of the small sample size. The salicoid WGD provided a major source of duplicated transcriptional regulators in *Populus*, and transcription factors and their interacting partners are often retained after WGD because of dosage-balance and regulatory-network constraints [27,28,29]. The relative enrichment of WGD/segmental duplicates in the core class therefore supports the view that some ancient bZIP duplicates were retained over long evolutionary periods and contributed to a stable family framework. The greater relative contributions of tandem and dispersed duplication to soft-core and shell genes may instead have facilitated lineage-associated copy-number variation among *Populus* genomes. However, the association between duplication mode and pan-genome class was modest (Cramér’s V = 0.239; sensitivity analysis, V = 0.218). These results support differences in the relative contributions of duplication modes to conserved and variable family components but do not identify any single mode as the sole cause of functional divergence.

Chromosomal distribution and local synteny in *P. trichocarpa* further suggest extensive redistribution of homologous segments following the salicoid WGD. No bZIP member was detected on *P. trichocarpa* Chr11 or Chr12, although members of the corresponding OGGs occurred on these chromosomes in other *Populus* genomes and were retained on other chromosomes in *P. trichocarpa*. Given the extensive chromosomal breakage, fusion and segmental reorganization that followed salicoid duplication in Salicaceae [30], this distribution is more consistent with relocation of homologous segments and differential retention among *Populus* lineages than with a demonstrated *P. trichocarpa*-specific loss of bZIP genes. Several loci excluded from the final bZIP set remained locally syntenic with confirmed bZIP genes despite showing reduced or undetectable PF00170 support. Across the species phylogeny, homologues of *Potri.010G128001* in Clade I consistently displayed partial or weak PF00170 evidence, whereas the homologues of *Potri.019G057550* lacking detectable PF00170 were distributed across Clades I and II. The two loci therefore do not support a common, single-lineage pattern of domain loss. Instead, their conserved genomic context and variable domain evidence may reflect post-duplication sequence divergence, although truncated or incomplete gene models could produce similar patterns [31]. These loci should therefore be considered candidates for further investigation rather than confirmed examples of asymmetric domain loss or inactive bZIP copies.

TE distributions further illustrated the complexity of the local genomic environments surrounding *Populus* bZIP genes. Overall, 72.2% of bZIP genes overlapped at least one TE within the gene body or 2-kb flanking regions, with TIR and LTR elements predominating. However, the TE-positive proportion of bZIP genes was modestly lower than that of matched non-bZIP genes, and copy-number-variable OGGs did not show greater aggregate TE coverage than invariant OGGs; the bootstrap confidence intervals for the corresponding effect sizes included zero. These results provide no evidence that preferential TE accumulation was a primary driver of bZIP family expansion or copy-number variation. Instead, the predominance of WGD/segmental duplication suggests that large-scale genome duplication generated most bZIP copies. Transcription-factor duplicates produced by WGD are often constrained by dosage balance and purifying selection and may therefore be retained over long evolutionary periods [28,32]. Nevertheless, because TE insertions can influence local chromatin states and gene regulation [33,34], variation in TE content may still contribute to locus-specific structural or regulatory divergence. The present overlap-based analysis does not establish such functional effects, which require locus-level regulatory evidence. Compared with the variability observed in their local genomic environments, bZIP coding sequences showed strong overall evidence of purifying selection. Among the retained comparisons, 97.6% had Ka/Ks ≤ 1, and the median Ka/Ks was 0.290. The consistently low OGG-level Ka/Ks values suggest that functional constraint has dominated bZIP evolution across *Populus*. Although selective pressure varied among subfamilies, the limited substitution information underlying some estimates and the lack of statistical support for nearly all Ka/Ks values above 1 argue against widespread positive selection [35]. The observed heterogeneity is therefore more plausibly interpreted as variation in the strength of purifying constraint, with possible localized relaxation in particular evolutionary lineages.

Against this genomic evolutionary background, multispecies RNA-seq analysis showed that coding-sequence conservation did not translate directly into a conserved transcriptional response to salt stress. Overall, significant responses were detected for 45 distinct bZIP genes from three of the five taxa with replicated contrasts. In *P. trichocarpa*, the sustained upregulation of *Potri.014G120800* at both treatment stages, together with the experimentally demonstrated role of its corresponding gene PagbZIP75 in ABA-associated salt tolerance and ROS regulation [10], provides convergent expression and functional evidence supporting this gene as a priority candidate. Of the 76 OGGs represented in the expression datasets, 29 responded significantly in only one taxon, whereas the three OGGs detected in two taxa showed opposite response directions. Previous comparative transcriptomic and metabolomic analyses have shown that salt-sensitive and salt-tolerant poplar species can recruit distinct stress-response pathways [36]. However, because the public datasets integrated here differed in genotype, salt regime, treatment duration, sequencing platform, and statistical power, the limited concordance observed in this study cannot be attributed unambiguously to biological divergence. Genes upregulated under prolonged treatment in *P. trichocarpa* also showed trends toward light-signaling and circadian-rhythm annotations, consistent with known roles of HY5/HYH in light signaling, circadian regulation, and salt-stress responses [24,37,38]. These trends did not remain significant after multiple-testing correction and should therefore be considered tentative candidate associations.

## 4. Materials and Methods

### 4.1. Identification and Pan-Genome Classification of Populus bZIP Genes

Genome assemblies and structural annotations for the 19 *Populus* genomes were obtained from NGDC/CNCB, Phytozome-JGI, PlantGenIE, GigaDB, and NCBI; assembly continuity, gene-set BUSCO completeness, and annotation provenance are summarized in Supplementary Table S8. Candidate bZIP proteins were identified independently in each genome using BITACORA v1.4 [39], with 127 *Arabidopsis thaliana* and 214 *P. trichocarpa* bZIP proteins from PlantTFDB [40] and the Pfam PF00170.27 profile [41] as references. Sequence-similarity and domain searches used an E-value threshold of 1 × 10^−5^, and candidates were initially required to contain a 21–65-aa region spanning the basic region and adjacent leucine zipper. PF00170 domains were reassessed using HMMER v3.4 [42]; a domain was classified as canonical when the full-sequence bit score reached the Pfam gathering threshold of 27.6 and the domain i-E-value was ≤1 × 10^−5^ (Supplementary Table S21). The BITACORA-derived candidate records were further audited for genomic coordinates, strand orientation, exon structure, ORF integrity, start and stop codons, internal stop codons, and overlap with existing transcript annotations. Incomplete models were reconstructed by protein-to-genome alignment with miniprot v0.18-r281 [43], initially using full-length bZIP proteins from the same preliminary OGG within a ±30-kb window. For unresolved loci, closely related full-length *Populus* bZIP proteins identified by BLASTP [44] were mapped within an expanded ±100-kb window. Recovered models were required to overlap the original locus on the same strand, cover ≥80% of the reference protein, contain a complete ORF, and encode a canonical PF00170 domain. Models recovered in the expanded search additionally required ≥50% sequence identity, ≥70% reciprocal overlap among reconstructions, and support from complete homologous models in at least two other *Populus* taxa. Models meeting these criteria were incorporated into the revised bZIP set, and their GFF3 annotations, CDS sequences, and protein sequences were provided in the Supplementary Data and Supplementary Table S1. To assess transcriptional support, the CDS sequences of the 25 retained models were searched against same-species NCBI EST, TSA, and submitted non-RefSeq mRNA/cDNA records using BLASTN in megablast mode with an E-value threshold of 1 × 10^−10^. Matches with ≥95% nucleotide identity and ≥50% query coverage were considered locus-level transcript evidence, whereas those with ≥80% coverage were classified as near-full-length support (Supplementary Table S1). Subsequently, orthologous gene groups (OGGs) were inferred using OrthoFinder v2.5.5 with the default MCL inflation value of 1.5 [45], using all-versus-all DIAMOND v2.2.2 searches in --more-sensitive mode with an E-value threshold of 1 × 10^−3^ [46]. When an OGG contained one or more *P. trichocarpa* members, the longest *P. trichocarpa* protein was selected as its representative; otherwise, the longest protein in the OGG was used. Representative sequences were renamed according to the pan-gene family nomenclature. OGGs present in 19, 17–18, 2–16, or 1 genome(s) were classified as core, soft-core, shell, or cloud, respectively (Supplementary Tables S4 and S5). Apparent absences in soft-core and shell OGGs were further assessed by searching the corresponding genome assemblies with the OGG representative proteins using TBLASTN; the E-value and query coverage of each best hit were recorded (Supplementary Table S7). As sensitivity analyses, OGG inference and pan-genome classification were rerun with identical settings after excluding either the 21 independent new loci alone or all 25 supplementary models. Proteins unassigned by OrthoFinder were reassessed using gene structure, PF00170 domain, homology-mapping, and transcriptomic evidence. Models with complete ORFs and canonical PF00170 domains were retained in the family dataset but excluded from OGG-based analyses when model replacement was not sufficiently supported (Supplementary Table S3).

### 4.2. Phylogenetic Classification and Conserved-Structure Analysis

Reference proteins from *Arabidopsis thaliana* and rice were obtained from Araport11 [47] and the Nipponbare MSU Release 7 proteome [48], respectively (Supplementary Table S22a). The reference proteins and OGG representatives from the 19 *Populus* genomes were aligned using MAFFT v7.505 in --auto mode [49], and alignment columns containing more than 80% gaps were removed. A maximum-likelihood phylogenetic tree was constructed using IQ-TREE v2.0.7 [50], with ModelFinder selecting the best-fit substitution model according to the Bayesian information criterion [51]. Branch support was evaluated using 1000 SH-aLRT replicates and 1000 ultrafast bootstrap replicates. Branches with SH-aLRT ≥ 80% and ultrafast bootstrap support ≥ 95% were considered strongly supported [52], and only nodes with ultrafast bootstrap support ≥ 95% were labelled in the tree. Subfamily assignments followed the 13-group classification framework proposed by Corrêa et al. [6]. The phylogenetic tree was visualized and annotated using iTOL. Ten conserved motifs were identified from the OGG representative proteins using MEME Suite v5.5.9 under the ZOOPS model with a zero-order background model estimated from the input sequences; 10 motifs were requested, with motif widths restricted to 6–50 amino acids [53]. The resulting motif models were then used to scan all *Populus* bZIP proteins with MAST v5.5.9, using a sequence E-value threshold of 10 and a motif-hit *p*-value threshold of 1 × 10^−4^. Motif coordinates were integrated with PF00170 domain positions and phylogenetic relationships, and motif occurrence frequencies were calculated for each subfamily (Supplementary Table S9a). Representative MAST matches and their sequence coordinates are provided in Supplementary Table S9b. To independently assess heptad periodicity in the leucine-zipper region of group D proteins, the *Populus* group D proteins and the corresponding *A. thaliana* and rice reference proteins were aligned to the PF00170.27 profile using HMMER v3.4 [42]. PF00170 match state 31 was designated as the first d position, and four subsequent positions at seven-residue intervals were extracted. A, F, I, L, M, V, W, and Y were classified as hydrophobic residues. The heptad periodicity score was defined as the difference between the proportion of hydrophobic residues at the fixed d positions and that at non-a/d positions within the same aligned region. These proportions were compared using a one-sided paired Wilcoxon signed-rank test, and 95% confidence intervals were estimated from 10,000 bootstrap resamples (Supplementary Tables S10 and S22b). An OGG was classified as copy-number-variable when its member count differed among the 19 *Populus* genomes and as copy-number-invariant when its member count was identical across all genomes (Supplementary Table S6). The number and proportion of copy-number-variable OGGs were subsequently calculated for each classified subfamily; unclassified OGGs were excluded from the subfamily-level summary. Experimental functional evidence for representative homologs from the different subfamilies was also compiled (Table 1; Supplementary Table S11).

### 4.3. Chromosomal Distribution and Evolutionary Analyses

Within-proteome all-versus-all BLASTP searches were conducted separately for the 19 proteomes using an E-value threshold of 1 × 10^−5^, and the five highest-scoring non-self hits per query were retained [44]. The resulting genome-specific BLASTP and GFF3 files were analysed using MCScanX v1.0.0 with its default settings (match score = 50, gap penalty = −1, E-value threshold = 1 × 10^−5^, and MATCH_SIZE = 5) to identify intragenomic synteny and classify genes as WGD/segmental, tandem, proximal, dispersed, or singleton duplicates [54]. Associations between duplication mode and pan-genome class were tested using a 4 × 5 Fisher–Freeman–Halton exact test with 1,000,000 Monte Carlo fixed-margin tables (seed = 20260721), and effect size was quantified using Cramér’s V. As a sensitivity analysis, cloud was combined with shell, while proximal, tandem, and singleton genes were grouped as local/other, and the resulting 3 × 3 table was evaluated using Pearson’s chi-square test (Supplementary Table S12a–c). The *P. trichocarpa* reference genome was used to examine chromosomal distribution and intragenomic synteny. Gene coordinates were obtained from the GFF3 annotation, and MCScanX relationships were visualized using Tbtool v2.487 [55]. A strict bZIP–bZIP syntenic pair was defined as an MCScanX relationship in which both endpoints belonged to the final bZIP gene set. Relationships containing one confirmed bZIP endpoint and one endpoint outside this set were analysed separately. For each candidate relationship, five flanking genes on either side of both loci were extracted for local microsynteny analysis. Candidate–anchor protein similarity was assessed using BLASTP, and anchor proteins were aligned to candidate genomic regions using TBLASTN to examine possible gene-model truncation or missing annotation [44]. Cross-species homologues of the candidate proteins were identified by BLASTP searches against the 19 *Populus* proteomes. Candidate proteins, their syntenic bZIP anchors, and the corresponding homologues were screened against PF00170.27 using HMMER [41,42]. Hits were classified as canonical when the full-sequence HMM bit score reached the Pfam gathering threshold (GA = 27.6), as partial when the score was below GA but the independent E-value was ≤1 × 10^−3^ and HMM coverage was ≥30%, as weak signal when the independent E-value was ≤1 and coverage was ≥30%, and as not detected otherwise. The family-wide PF00170 inclusion threshold was E ≤ 1 × 10^−5^. PF00170 scores and states were mapped onto the published phylogeny of the 19 *Populus* taxa [3], retaining its original topology and branch lengths while adding only the HMM-score and domain-state annotations shown in Figure 4e (Supplementary Table S13a–c).

Transposable elements in the 19 *Populus* genomes were identified using HiTE v3.3.3 and annotated using RepeatMasker v4.1.1 with the genome-specific consensus TE libraries generated by HiTE [56] (Supplementary Table S14c). Only high-confidence, classified TEs were retained; simple repeats, low-complexity sequences, satellites, and RNA-derived repeats were excluded. For each bZIP gene, the selected mRNA span, including annotated UTRs, and its 2-kb upstream and downstream regions were analyzed. Overlapping TE intervals within each window were merged before coverage calculation. TEs were grouped into TIR, LTR, non-LTR, and Helitron classes while retaining subclass annotations. TE-covered fractions among pan-genome classes were compared using the Kruskal–Wallis test (Supplementary Table S14a). For background comparison, each bZIP gene was matched by genome, chromosome, transcript length, and relative chromosomal position to a non-bZIP gene; this procedure was repeated 1000 times (Supplementary Table S14b). Copy-number-variable and invariant OGGs were compared using a two-sided Mann–Whitney U test, with Cliff’s delta and its 95% confidence interval estimated from 50,000 bootstrap replicates (Supplementary Table S14d,e).

To assess selection pressure among homologous *Populus* bZIP genes, anchor-to-member comparisons were constructed within each OGG. Protein alignments and the corresponding codon alignments were generated using ParaAT v2.0 [57], and Ka, Ks, and Ka/Ks were estimated using the YN00 method [58] implemented in KaKs_Calculator v1.2 [59]. Comparisons with missing, undefined or nonfinite estimates were excluded. Following previously applied quality control criteria [60], comparisons with Ks < 0.01, Ks > 2, Ka > 2 or Ka/Ks > 10 were further excluded to reduce the effects of near-zero Ks values, substitution saturation and potentially unreliable parameter estimates (Supplementary Table S15a,b). Statistical support was evaluated using the Fisher test reported by KaKs_Calculator, and *p*-values for all retained comparisons were adjusted using the Benjamini–Hochberg procedure, with an adjusted *p* < 0.05 considered significant. To avoid pseudoreplication, retained pairwise estimates were summarized as the median Ka/Ks for each OGG before group-level comparisons. OGG-level medians were compared between core and non-core OGGs using a two-sided Mann–Whitney U test, with Cliff’s delta and its 95% bootstrap confidence interval reported as the effect size, and among the 13 classified subfamilies using a Kruskal–Wallis test (Supplementary Table S15c,d). Pairwise estimates were retained only for descriptive visualization and Fisher tests; estimates based on fewer than 10 substitutions were flagged as having limited information content but were not excluded.

### 4.4. Salt-Stress-Responsive Expression Analysis

Salt-stress RNA-seq datasets from leaf tissues of six *Populus* taxa were downloaded from the NCBI Sequence Read Archive (SRA) [61]: *P. alba* var. *pyramidalis* (SRP116769), *P. simonii* (SRP095916), *P. yunnanensis* (SRP563339), *P. trichocarpa* (ERP021848), *P. deltoides* (SRP431143), and *P. euphratica* (ERP121336) (Supplementary Table S16). The ERP121336 study includes both root- and leaf-derived RNA-seq libraries; only the 20 leaf libraries were included in the present analysis. Adapters and low-quality reads were removed using fastp v0.23.4, which was also used to generate quality control reports [62]. Filtered paired-end reads were aligned to the corresponding reference genomes using HISAT2 v2.2.2 in --dta mode [63], and the resulting alignments were sorted and indexed using SAMtools v1.23.1 [64]. For each taxon, GFF3 records for the retained supplementary models from the corresponding genome were appended to the original structural annotation. CDS intervals lacking explicit exon records were represented as exon features in the counting GTF. Gene-level counts were obtained with HTSeq-count v2.1.2 in union mode using unstranded counting, exon features, and the gene_id attribute [65]. TPM values were also calculated for expression summaries and assessment of transcriptional support for the retained supplementary models (Supplementary Table S17a,b). A retained supplementary bZIP model was considered to have detectable transcription when TPM > 0 in at least one library; TPM ≥ 1 was additionally reported as a more conservative descriptive threshold for transcript abundance. For contrasts with biological replicates, DESeq2 v1.50.2 was used for count normalization, dispersion estimation, and differential-expression testing [66] (Supplementary Table S19d). Genes with an absolute log2 fold change ≥ 1 and a Benjamini–Hochberg-adjusted *p*-value ≤ 0.05 were defined as significantly salt responsive (Supplementary Table S18). The *P. yunnanensis* dataset lacked biological replicates suitable for estimating within-group variance; therefore, only TPM-based fold changes and response directions were calculated. For cross-taxon summaries, an OGG was considered responsive in a taxon when at least one mapped member showed a significant response in a replicated contrast. Response direction was assigned as up- or downregulated only when all significant member–contrast responses were concordant; otherwise, the OGG was classified as mixed (Supplementary Table S19a). GO and KEGG annotations were generated using InterProScan v5.78-109.0 (InterPro 109.0) and KofamScan v1.3.0 (KOfam database, June 2026), respectively (Supplementary Table S19b). Functional-annotation proportions were then calculated for the Gene Ontology (GO) biological process, molecular function and cellular component domains and for the selected Kyoto Encyclopedia of Genes and Genomes (KEGG) pathways using the corresponding taxon-level bZIP set as the denominator. For each replicated contrast, GO and KEGG over-representation was evaluated separately for the up- and downregulated bZIP sets using a one-sided hypergeometric test implemented in Python v3.13.7. All bZIP genes with finite log2 fold-change estimates in the corresponding contrast were used as the background. Analyses were restricted to response sets containing at least five genes, and only terms represented by at least two responsive genes were tested. *p*-values were adjusted using the Benjamini–Hochberg method, with adjusted *p* < 0.05 considered statistically significant (Supplementary Table S19c).

## 5. Conclusions

At the genus level, the *Populus* bZIP family exhibited a broadly conserved genomic framework accompanied by substantial variation among the 19 genomes. Across the 19 genomes, 1764 bZIP proteins were identified, of which 1762 were assigned to 79 OGGs, with most members occurring in orthogroups broadly retained across the genus. Copy-number variation was nevertheless widespread within shared OGGs, indicating that family diversity arose through both differential orthogroup retention and lineage-dependent changes in member number. The predominance of WGD/segmental duplicates and Ka/Ks values below 1 supports an important role for ancient duplication followed by purifying selection in maintaining this conserved framework. Although TEs were common in bZIP gene regions, no preferential TE accumulation associated with family expansion or copy-number variation was detected. Across the heterogeneous public salt-stress datasets analyzed, no OGG showed a significant, directionally concordant response in at least two *Populus* taxa; consequently, the extent of cross-taxon conservation in bZIP salt responses remains unresolved. Collectively, this study provides a genus-wide framework for understanding the conservation and diversification of the *Populus* bZIP family and establishes a comparative foundation for future functional studies under standardized experimental conditions.

## Figures and Tables

**Table 1:** Gene counts, copy-number-variable OGGs, and representative functions across *Populus* bZIP subfamilies.

Subfamily	Genes, *n*	CNV OGGs, *n*/Total (%)	Representative Genes	Function	Supporting Reference ID(s)
A	361	12/17 (70.6%)	*AtABF2/AREB1; AtABI5; OsbZIP23*	Mediates ABA-dependent transcription during seed germination and post-germinative growth arrest; regulates glucose signaling and improves drought, salinity, and other abiotic-stress responses.	SR1, SR2, SR3, SR4
B	39	1/2 (50%)	*AtbZIP28; AtbZIP17; OsbZIP39*	Functions as a membrane-associated stress-sensing module: regulated proteolysis and nuclear relocation activate endoplasmic reticulum and heat-stress responses, with links to brassinosteroid-dependent stress acclimation.	SR5, SR6, SR7, SR8
C	105	4/6 (66.7%)	*AtbZIP63; AtbZIP10; OsbZIP58*	Coordinates low-energy signaling through SnRK1-dependent dimerization; also contributes to basal defense and cell-death control, while cereal homologs regulate endosperm starch biosynthesis.	SR9, SR10, SR11
D	255	8/12 (66.7%)	*AtTGA3; AtTGA9/TGA10; PERIANTHIA; rice TGA factors*	Integrates salicylic-acid/NPR1-dependent defense transcription and disease resistance; selected members additionally control anther development and floral-organ number.	SR12, SR13, SR14, SR15
E	57	2/3 (66.7%)	*AtbZIP34*	Controls pollen-wall patterning and influences lipid-metabolism and cellular-transport pathways during pollen development.	SR16
F	74	4/4 (100%)	*AtbZIP19/AtbZIP23; AtbZIP24*	Regulates adaptation to zinc deficiency and broader abiotic-stress transcriptional networks, including salt-stress responses.	SR17, SR18
G	114	5/5 (100%)	*AtbZIP16; AtbZIP68; AtGBF1*	Regulates blue-light-dependent photomorphogenesis and the establishment of photosynthetically active chloroplasts through redox-sensitive control of nuclear photosynthesis genes.	SR19, SR20
H	56	2/2 (100%)	*AtHY5; AtHYH*	Acts downstream of COP1 in light-dependent gene expression and photomorphogenesis; HY5 also coordinates light and copper homeostasis through the HY5-SPL7-miR408 network.	SR21, SR22
I	161	3/5 (60%)	*OsRF2a; AtVIP1; AtbZIP18*	Includes regulators of vascular development, MAPK-responsive stress transcription, and male-gametophyte development.	SR23, SR24, SR25
J	18	1/1 (100%)	*AtbZIP62*	Contributes positively to drought acclimation and regulates drought-associated nitrate transport and assimilation; mutant evidence also suggests negative regulation of basal defense and systemic acquired resistance.	SR26, SR27, SR28
K	19	0/1 (0%)	*AtbZIP60; OsbZIP74*	Mediates the IRE1-dependent unfolded-protein response through stress-induced unconventional mRNA splicing, linking endoplasmic reticulum homeostasis with heat, abiotic-stress, and immunity responses.	SR29, SR30, SR31
L	79	4/4 (100%)	*AtbZIP77 (AT1G35490); OsbZIP21; OsbZIP82*	Direct functional evidence for subfamily L remains limited. Available data suggest potential roles in male reproductive development and environmental stress responses.	SR32, SR33
S	357	9/12 (75%)	*AtbZIP1/AtbZIP53; AtbZIP11; AtbZIP44*	Reprograms amino-acid and central metabolism under low-energy or sucrose signals; selected members regulate seed maturation, germination, and cell-wall reserve mobilization.	SR34, SR35, SR36, SR37

Note: An OGG was classified as copy-number-variable when its member count differed among the 19 *Populus* genomes. Gene counts represent all genes contained in OGGs assigned to each subfamily. CNV values indicate the number of copy-number-variable OGGs relative to the total number of OGGs assigned to each subfamily. The 100% CNV values for subfamilies F, G, H, J, and L are based on 4, 5, 2, 1, and 4 OGGs, respectively, and should be interpreted in light of these small denominators. Five unclassified OGGs comprising 67 genes and two proteins not assigned to any OGG were excluded from the subfamily-level summary. SR1–SR37 denote supporting literature references for the representative homologs and functional evidence summarized in Table 1; full bibliographic details are provided in Supplementary Table S11.

## Data Availability

The complete custom analysis scripts used in this study, together with usage instructions, software dependencies, representative input data, script manifests, checksums, retained gene models, orthogroup assignments, and supporting source data, are available on GitHub (https://github.com/QILIN0207/populus-bzip-analysis; accessed on 24 August 2026) and archived in Zenodo at https://doi.org/10.5281/zenodo.21976782. The remaining data supporting the findings of this study are provided in the article and its Supplementary Materials.

## Supplementary Materials

The following supporting information can be downloaded at https://www.mdpi.com/article/10.3390/plants15172600/s1: Table S1: Structural audit, recovery evidence, and final classification of 52 BITACORA-derived *Populus* bZIP candidate records; Table S2: Full-length protein sequences of 1764 *Populus* bZIP proteins; Table S3: Structural, homology-mapping, and transcript-evidence audit of two *Populus* bZIP proteins not assigned to orthogroups; Table S4: Membership and pan-genome classification of 79 *Populus* bZIP orthogroups; Table S5: Summary statistics, representative pangene IDs, subfamily assignments, and member sources for 79 *Populus* bZIP orthogroups; Table S6: Copy-number matrix of *Populus* bZIP orthogroups across 19 genomes; Table S7: Assembly-level TBLASTN audit of apparent absence calls in soft-core and shell *Populus* bZIP orthogroups; Table S8: Genome assembly, annotation quality, and data sources for the 19 *Populus* genomes; Table S9a: Occurrence frequencies and sequence logos of MEME motifs across *Populus* bZIP subfamilies; Table S9b: Representative MAST matches for the 10 MEME motifs in *Populus* bZIP proteins; Table S10: PF00170-anchored assessment of heptad periodicity in group D bZIP proteins; Table S11: Supporting literature for the functional annotations and comparative interpretation of bZIP subfamilies summarized in Table 1; Table S12a: Duplication modes of 1762 OGG-assigned *Populus* bZIP proteins; Table S12b: Summary of duplication modes by pan-genome class; Table S12c: Association between duplication mode and pan-genome class; Table S13a: bZIP loci located on chromosomes 11 and 12 in *Populus* genomes other than the *P. trichocarpa* reference genome; Table S13b: Weak PF00170 hits on *P. trichocarpa* chromosomes 11 and 12; Table S13c: Synteny, domain status, and cross-species evidence for atypical bZIP-related loci in *P. trichocarpa*; Table S14a: Individual transposable-element overlaps and gene-level TE coverage for *Populus* bZIP loci; Table S14b: Comparison of TE overlap between *Populus* bZIP genes and matched non-bZIP background genes; Table S14c: Per-genome HiTE/RepeatMasker annotation summaries; Table S14d: Statistical comparison of TE coverage between copy-number-variable and invariant *Populus* bZIP orthogroups; Table S14e: OGG-level TE coverage metrics for copy-number-variable and invariant *Populus* bZIP orthogroups; Table S15a: Filtered within-orthogroup Ka, Ks, and Ka/Ks estimates for *Populus* bZIP gene pairs; Table S15b: Excluded within-orthogroup *Populus* bZIP comparisons and explicit exclusion reasons; Table S15c: OGG-level summaries of the 2231 filtered within-orthogroup *Populus* bZIP comparisons; Table S15d: Statistical tests for the *Populus* bZIP Ka/Ks analysis; Table S16: Transcriptome data sources, sample metadata, and per-run sequencing quality for multispecies salt-stress analyses; Table S17a: RNA-seq expression and public-transcript support for the 25 retained supplementary *Populus* bZIP models; Table S17b: Per-sample TPM values for retained supplementary *Populus* bZIP models evaluated in the available RNA-seq datasets; Table S18: Differential-expression statistics and salt-response classifications of bZIP genes across six *Populus* taxa; Table S19a: Cross-taxon salt-response summary for *Populus* bZIP orthogroups; Table S19b: GO and KEGG functional-annotation profiles; Table S19c: GO and KEGG over-representation results; Table S19d: Quality control summary for sequencing, biological replication, expression testing, and replicate correlations across RNA-seq contrasts; Table S20: *Arabidopsis* and *Populus* bZIP full-length protein sequences downloaded from PlantTFDB; Table S21: PF00170.27 HMMER domain-search results for 1764 *Populus* bZIP proteins across 19 genomes; Table S22a: *Arabidopsis* and rice bZIP full-length protein sequences used as phylogenetic references; Table S22b: PF00170 domain sequences extracted from the *Arabidopsis* and rice bZIP phylogenetic references; Supplementary Data: GFF3 annotations, CDS sequences, and protein sequences for the 25 retained supplementary *Populus* bZIP models.

## Abbreviations

ABA	Abscisic acid
ABRE	ABA-responsive element
AREB/ABF	ABA-responsive element-binding protein/ABRE-binding factor
bZIP	Basic leucine zipper
CNV	Copy number variation
DNA	Deoxyribonucleic acid
GA	Pfam gathering threshold
GFF3	Generic Feature Format version 3
GO	Gene Ontology
HMM	Hidden Markov model
HY5/HYH	Elongated Hypocotyl 5/HY5 Homolog
JTT	Jones-Taylor-Thornton amino acid substitution model
Ka	Nonsynonymous substitution rate
Ka/Ks	Ratio of nonsynonymous to synonymous substitution rates
KEGG	Kyoto Encyclopedia of Genes and Genomes
Ks	Synonymous substitution rate
LTR	Long terminal repeat
MAPK	Mitogen-activated protein kinase
NCBI	National Center for Biotechnology Information
non-LTR	Non-long-terminal-repeat retrotransposon
NPR1	Nonexpressor of Pathogenesis-Related Genes 1
OGG	Orthologous gene group
PAV	Presence/absence variation
PlantTFDB	Plant Transcription Factor Database
RNA-seq	Ribonucleic acid sequencing
SH-aLRT	Shimodaira-Hasegawa-like approximate likelihood-ratio test
SnRK2	Sucrose nonfermenting 1-related protein kinase 2
SRA	Sequence Read Archive
TE	Transposable element
TGA	TGACG motif-binding factor
TIR	Terminal inverted repeat
TPM	Transcripts per million
WGD	Whole-genome duplication
